# The lifespan-extending MEK1 inhibitor trametinib promotes regulation of de novo lipogenesis enzymes by chaperone-mediated autophagy

**DOI:** 10.3389/fragi.2025.1621808

**Published:** 2025-06-25

**Authors:** Jiexian Chen, Joshua Berg, Calvin M. Burns, Hanyi Jia, Xinna Li, Richard A. Miller, S. Joseph Endicott, Gonzalo Garcia

**Affiliations:** ^1^ Department of Chemical Biology, University of Michigan College of Literature, Science, and the Arts, Ann Arbor, MI, United States; ^2^ Department of Pathology, University of Michigan School of Medicine, Ann Arbor, MI, United States; ^3^ Department of Molecular, Cell, and Developmental Biology, University of California, Los Angeles, Los Angeles, CA, United States; ^4^ Geriatrics Center, University of Michigan, Ann Arbor, MI, United States; ^5^ Department of Pathology, University of New Mexico, Albuquerque, NM, United States; ^6^ Autophagy, Inflammation, and Metabolism (AIM) Center, University of New Mexico, Albuquerque, NM, United States

**Keywords:** aging, extracellular signal-regulated kinase (ERK), chaperone-mediated autophagy (CMA), signal transduction, transcriptional response, lifespan, longevity

## Abstract

The availability of multiple slow-aging mice allows a search for possible shared mechanisms that affect the rate of aging. Previous work has shown downregulation of the MEK1-ERK-MNK kinase cascade, which regulates protein translation through eIF4E, in response to four anti-aging drugs. Here we show that decreased protein abundance of enzymes involved in hepatic *de novo* lipogenesis (DNL) is characteristic of mice exposed to two anti-aging drugs that modulate glucose homeostasis (acarbose and canagliflozin), as well as in calorically restricted mice and in two long-lived mutant models. The same pattern of changes in the *de novo* lipogenesis enzymes can be produced, in cultured cells or in intact mice, by trametinib, a drug that inhibits the MEK-ERK kinase cascade, and which has been shown to extend mouse lifespan. The trametinib effect on DNL enzymes is, unexpectedly, not related to transcriptional changes, but depends on selective protein degradation through chaperone-mediated autophagy. Our data support models in which chaperone-mediated proteomic alterations, triggered through the MEK1-ERK-MNK kinase pathway, may collaborate with mTORC1 changes to slow aging and extend mouse lifespan.

## Introduction

Recent work has shown that mouse lifespan can be extended by at least five single-gene mutations (Brown-Borg et al., 1996; Coschigano et al., 2000; Flurkey et al., 2001; Conover and Bale, 2007; Ortega-Molina et al., 2012), at least two dietary interventions (Masoro et al., 1982; Miller et al., 2005), and at least 8 drugs (Harrison et al., 2009; Harrison et al., 2014; Miller et al., 2020; Harrison et al., 2023; Miller et al., 2024). In the best-studied cases, data show that the lifespan extension is accompanied by, and presumably caused by, delay in many age-dependent forms of pathology (Wilkinson et al., 2012; Snyder et al., 2022; Conover et al., 2010; Maeda et al., 1985). These slow-aging mouse models provide tools for seeking shared mechanisms, i.e., changes in cellular or physiological state that may link the intervention to enduring health preservation. A recent review (Miller et al., 2023) provides a preliminary listing of biochemical and cellular changes that are characteristic of 10 of these varieties of slow-aging mice.

A growing set of published studies has sought commonalities among anti-aging mechanisms by comparative transcriptomics (Debes et al., 2023; Li et al., 2023; Tyshkovskiy et al., 2023; Tyshkovskiy et al., 2019). The underlying concept is that shared patterns of differential transcription could draw attention to pathways that might link apparently different interventions to possible shared mechanisms of slower aging and disease prevention. In principle, such shared transcriptomic signatures could provide a foundation for prioritizing candidate agents to favor those whose transcriptional profiles resemble that of authenticated anti-aging drugs (Duan et al., 2020).

There are now hints, however, that reliance on changes in transcription patterns may not be an ideal strategy for understanding mechanisms of action of anti-aging interventions and seeking new ones. Age-associated changes in mRNA abundance either do not correlate or correlate only weakly with age-associated changes in protein abundance in every system carefully examined to date, including the mouse kidney (Takemon et al., 2021), the mouse heart and skeletal muscle (Han et al., 2021) the mouse liver (Williams et al., 2022), the rat brain and liver (Ori et al., 2015), and human male skeletal muscle (Gueugneau et al., 2021). Taking mouse kidney as a specific example, only about 10% of protein changes that occur with age are concordant with changes in the corresponding mRNA (Takemon et al., 2021). This suggests that non-transcriptional processes are responsible for a very large fraction of age-associated changes in protein levels. In addition, the uncoupling between RNA levels and protein levels during aging suggests regulation of translation can play an important role in the aging process (Solyga et al., 2024).

Our laboratory has recently drawn attention to two non-transcriptional processes that are elevated in multiple varieties of slow-aging mice and mold the proteome in these animals. A subset of mRNAs can be translated by a cap-independent translation process (CIT), leading to upregulation of the corresponding proteins without parallel upregulation in the mRNA levels themselves. The mechanism of this preferential translation can be complex (Merrick, 2004; Leppek et al., 2018). In some cases CIT depends on ribosomal binding to 6-methyl-adenosine residues in the 5-prime untranslated region of a subset of mRNAs, leading to upregulation of the corresponding proteins without parallel upregulation in the mRNA levels themselves (Zhao et al., 2017). CIT is elevated in liver, muscle, and kidney of Snell dwarf, GHRKO, and Ames dwarf mice, as well as in mice treated with Rapamycin (Rapa), Acarbose (Aca), Canagliflozin (Cana), or 17α-estradiol (17aE2) (Li et al., 2022; Shen et al., 2021; Ozkurede et al., 2019). The lifespan benefits of Cana and 17aE2 are limited to male mice, and the effects of these drugs on CIT are also, intriguingly, male-specific (CIT). CIT is also elevated in mice given a diet low in Isoleucine, which leads to lifespan extension (Yeh et al., 2024). Phosphatidylinositol-glycan-specific phospholipase D1, a molecule induced by exercise and able to improve cognition and biochemical indices of brain health (Horowitz et al., 2020), is elevated in many slow-aging mouse models via CIT, rather than by transcriptional control (Li et al., 2022).

mRNA-specific translation may also be affected by phosphorylation status of eIF4E (Mahalingam and Cooper, 2001; Yang et al., 2020), whose phosphorylation ratio is downregulated in several varieties of slow aging mice, linked to downregulation of the MEK-ERK1/2 kinase cascade (Wink et al., 2022; Jiang et al., 2023). peIF4E levels are thought to regulate levels of enzymes involved in lipid metabolism (Conn et al., 2021), although the effect has so far been seen in studies of high-fat diets and related stress conditions rather than in mice on standard diets.

A third example of non-transcriptional modulation of the proteome emerged from studies of chaperone-mediated autophagy (CMA). CMA is a highly selective form of lysosomal proteolysis in which proteins bearing consensus motifs are individually selected by the chaperone Hspa8, which participates in their translocation into the lysosome for degradation (Cuervo et al., 1997; Dice, 1990). Unlike other forms of autophagy, CMA does not require vesicle trafficking or fusion events. Instead, CMA involves direct translocation of cytosolic proteins into the lysosomal lumen, dependent upon the lysosomal transmembrane protein LAMP2A (Cuervo and Dice, 1996). CMA is elevated in Snell dwarf and GHRKO mice (Endicott et al., 2021), as well as in the long-lived PTEN overexpression model (Zhang et al., 2023). Caloric restriction of rats and mice also leads to constitutively active CMA (Jafari et al., 2024). Our study of GHRKO mice found that only a small fraction of CMA-sensitive proteins, i.e., proteins whose transport to lysosomes depended on CMA, were actually diminished in GHRKO liver relative to their level in liver of littermate control mice (Endicott et al., 2022). A catalogue of 91 such proteins, i.e., those whose lower abundance in liver tissue of GHRKO mice was the result of CMA, included 16 ribosomal subunits, 4 proteins involved in initiation and elongation of protein translation, and at least 3 enzymes involved in synthesis of lipids and production of the metabolic intermediate acetyl-CoA (Endicott et al., 2022). CMA suppresses glycolysis, inhibits fatty acid synthesis, and reduces lipid droplet accumulation in cultured cells, reviewed by (Kaushik and Cuervo, 2018). These data collectively suggest a hypothetical mechanism, wherein lifespan extending interventions that reduce circulating insulin and IGF1, such as Snell (Papaconstantinou et al., 2005), GHRKO (Al-Regaiey et al., 2005), Phosphatidylinositol 3,4,5-trisphosphate 3-phosphatase and dual-specificity protein phosphatase (PTEN (Garc et al., 2012)), and calorie restriction, activate CMA to participate in transcription-independent remodeling of the proteome, which in turn confers unique longevity-promoting metabolic benefits (Endicott and Miller, 2024).

The insulin receptor and IGF1 receptor are both receptor tyrosine kinases (RTKs) that initiate signaling cascades upon ligand binding. This signaling cascade bifurcates into two arms, i.e., the PI3K/AKT pathway and the MEK/ERK pathway. We have shown that the PI3K/AKT pathway inhibits CMA, and inhibitors of class I PI3K activate CMA in cell culture and in mice (Endicott et al., 2020). However, the role of the MEK/ERK pathway in CMA regulation has not been previously characterized. The MEK/ERK pathway involves phosphorylation of MEK1, its targets ERK1 and ERK2, their targets MNK1 and MNK2, and eventually phosphorylation of eIF4E, an initiation factor for protein translation. We have shown (Wink et al., 2022; Jiang et al., 2023) that aging leads to increases in the phosphorylation level of each element in the MEK/ERK cascade, indicating elevation of the activity of each enzyme in the pathway, and that this elevation is inhibited by Rapa and Aca in both male and female mice, and by Cana and 17aE2 in male mice only. The congruence between sex-specificity in lifespan effects and the alterations in the MEK/ERK cascade suggests that the kinase changes are closely related to the lifespan effects, through pathways still to be elucidated.

A recent paper from the group of Linda Partridge (Xu et al., 2014) showed that trametinib (“Tram”), an inhibitor of MEK1, can extend mean and maximum lifespan of C3B6F1 hybrid mice of both sexes when provided to the mice in their food. Furthermore, Tram in combination with Rapa led to a lifespan extension larger than that seen in mice given either agent alone. These results imply that inhibition of the MEK/ERK cascade can, by itself, lead to extension of lifespan in at least one mouse stock and that the mechanism of extension may be distinct, at least partly, from inhibition of mTORC1 function.

In this paper we show that inhibition of the MEK/ERK cascade, by Tram, diminishes at least five enzymes involved in hepatic *de novo* lipogenesis (DNL), and does so via a non-transcriptional process. We show that Tram treatment enhances CMA in mouse liver and cultured cells, and that augmented CMA is sufficient to explain transcription-independent downregulation of DNL enzymes. Levels of these DNL enzymes are also downregulated in young adult mice by Aca, Cana, and the CR diet. CMA-mediated alteration in protein levels may be an important non-transcriptional regulator shared by several models of slower aging in mice, and may play a role in lifespan extension induced by Tram on its own or in combination with Rapa.

## Methods

### Antibodies

Commercially available antibodies are listed below.

ACACA/ACC1 (Cell Signaling Technology (CST): 4190S), ACLY (AbCam: 40793), ACSS2, ACTB/β-Actin (CST: 8457L), pAKT (CST: 4691S), AKT pS473 (CST: 4060S), CTSB (CST: 31718S), ENO1 (CST: 3810S), EEF1B2 (AbCam: ab228642), EEF1G (AbCam:ab72368), EEF2 (CST: 2332S), ERK1/2 (CST:4695S), ERK1/2 pT202/204 (CST: 4376S), FASN (AbCam: 22759), GAPDH (CST: 2118S), GFAP (AbCam: 7260), pS8 GFAP (Thermo: PA5-12991), H3 (AbCam: 176842), HSPA8/Hsc70 (AbCam: 154415), LAMP2A (AbCam: 125068), MAP1LC3B/LC3 (CST: 2775S), MAPT (CST: 46687S), ME1 (AbCam: ab97445), SQSTM1/p62 (CST: 5114S), MEK1 (CST:12671), pMEK1/2 pS217/221 (CST:9154), MNK1 (CST:2195), MNK2 (PTech:17354), pMNK pT197/202 (Inv:700242), eIF4E (CST:2067), peIF4E pS209 (Boster:P00135), S6 (CST:2217), pS6 pS235/236 (CST:4857), 4EBP1 (CST:9644), p4EBP1 pT37/46 (CST:2855), NDRG1 (CST:9408), pNSRG1 pT346 (CST:5482).

### Cell culture and trametinib-treated mice

For all cell culture experiments, cells were passaged not more than 20 times, after thawing the original stock obtained from ATCC. Cells were checked for *mycoplasma* contamination upon thaw, and were negative throughout this study. All cells were maintained in a humidified 37°C incubator with 10% CO2 and ambient O2. Recipes for cell culture growth media were obtained from the ATCC website. AML12 cells were obtained from ATCC (CRL-2254) and maintained in DMEM:F12 (Gibco: 11320033), supplemented with insulin-selenium-transferrin (Gibco: 51500-056), 10% FBS (Corning: 35-016-CV), 40 ng/mL dexamethasone (Sigma: D4902), and penicillin-streptomycin (Gibco: 15070063).

Mouse treatments, Snell Dwarf, GHRKO and respectively liver samples, proteomic datasets and data preprocessing have been previously described (Watanabe et al., 2023). For trametinib treated mice: five-month-old UM-HET3 mice were fed for 45 days with control 5LG6 diet (TestDiet.com) or 5LG6 diet containing 2.5 ppm of trametinib (InvivoChem:V0446)). Liver samples for Western blot and qRTPCR corresponding to Figures 2–4 were processed and analyzed as previously described (Wink et al., 2022; Jiang et al., 2023). Antibodies and qRTPCR probes are described in Supplementary Table S1.

### CMA reporter and microscopy

AML12 cells stably expressing the Dendra2 reporter were generated in our previous study (Endicott et al., 2020), and frozen aliquots of the same cells were used here. These cells were seeded in 24-well plates with a cover-glass (Fisher: 1254580) dropped into each well. Cells were treated as indicated on the figures. For fixation, cells were rinsed once with PBS, and fixed in a solution of 1% paraformaldehyde, 1% sucrose, in PBS, pH = 7.2, for 7 min at room temperature. Cells were rinsed twice with PBS, stained with Hoechst (Sigma: 94403), and mounted onto microscope slides (Fisher: 12-550-343), using fluorescent protein mounting medium (GBI Labs: E18-18). Slides were dried overnight at 4°C and were imaged the next day.

Microscopy was performed in the UM BRCF Microscopy Core. Images were acquired with a Zeiss Axioplan2 microscope equipped with Zeiss ApoTome for optical sectioning. Image capture was performed with a Zeiss AxioCam MRm camera. The objective lens used was a Zeiss Plan-NEOFLUAR ×40, with a numerical aperture of 0.75 (this lens requires no immersion medium). The microscope was operated with Zeiss Axiovision software. All microscopy was performed on fixed samples at room temperature. For each experimental replicate, equal numbers of images for each experimental group were acquired on the same day, using the exact same exposure settings and light intensity. Image stacks were acquired with a 0.5 μm z-plane slice-distance. Fluorescence quantification was performed in ImageJ, using raw, unadjusted images. Post-acquisition image adjustment was only performed for display images (i.e., only after analysis was complete), using ImageJ software.

Dendra2 puncta were counted blind, with images analyzed in random order, using our previously published methods (Endicott et al., 2020). First, images were subjected to the ImageJ Max Entropy thresholding algorithm to identify cells with sufficient fluorescent protein expression to be counted. Then the number of puncta per cell were manually counted in unadjusted images and recorded (because of uneven background, automated counting is consistently less accurate than manual counting). Cells were excluded from analysis if they were on the edge of the image (such that a portion of the cytosol was outside of the image, preventing an accurate count), or if their cytoplasm overlapped substantially with another cell, preventing accurate quantification. Final results were un-blinded and tabulated by a different person than the one performing the analysis. For each experiment, data were pooled from at least three independent replicates.

### Drugs for cell culture experiments

Drugs for cell culture experiments were obtained as follows: Bafilomycin A (Sigma; SML1661), Trametinib (InvivoChem: V0446), Leupeptin (Sigma; L2884), Ammonium Chloride (Sigma: A4514).

Bafilomycin A was used at a concentration of 160 nM for all experiments. All other drug doses and incubation times are as indicated in the figure legends or directly on the figures. Bafilomycin A was dissolved in DMSO (Sigma; D2650). Leupeptin (Sigma; L2884) was solubilized in sterile PBS.

For all drug treatments, the cells were maintained in complete growth medium, with serum, according to the recipes specified above.

### Lysosome isolation–light and heavy lysosomes

Mice were dissected at approximately the same time for each experiment (between 9 and 10 a.m., with the dark period ending at 6 a.m.). All mice were allowed free access to food during the entire course of experimentation. Freshly dissected liver tissue was immediately washed in ice-cold PBS, until no visible blood remained. All subsequent steps were performed at 4°C, and were carried out according to our published protocol (Burns C. M. et al., 2024). Tissue was diced with scissors in ice-cold 0.25M sucrose (Fisher; 57-50-1), pH = 7.2. The diced tissue was then gently homogenized in a Wheaton dounce homogenizer (Wheaton: 357538) with the “LOOSE” pestle (0.089-0.14 mm gap). The homogenate volume was adjusted to 2 mL with ice-cold 0.25 M sucrose, before a 3-min centrifugation at 6,800 × g to remove unbroken cells, extracellular matrix, and nuclei. The supernatant was collected and spun at 17,000 x g for 10 min to pellet the remaining organelles, which include the lysosomes. The pellet was resuspended in 0.25 M sucrose to a volume of 800 µL and then gently mixed with 1,600 µL of 88.38% Histodenz (Sigma, D2158). The sample was loaded into an ultracentrifuge tube (Beckman Coulter, 344057) for a 55Ti-SW rotor (Beckman Coulter, 342194). Four layers were laid over the sample: 0.8 mL 33.74% Histodenz, 0.8 mL 26.99% Histodenz, 0.8 mL 20.25% Histodenz, 0.4 mL 0.25 M sucrose. The samples were subjected to ultracentrifugation at 141,000 x g for 2 h in a Beckman Coulter L-70 ultracentrifuge. During ultracentrifugation, the sample resolved into visible bands at the interfaces of the density layers. The visible bands at the interface of the 0.25 M sucrose-20.25% Histodenz layers and the interface of the 20.25%–26.99% Histodenz layers were both highly enriched in lysosome markers, such as LAMP2A and CTSD, and were referred to as “light” and “heavy” lysosomes, respectively. After the lysosome-containing bands were removed from the density gradient, the sample were diluted in PBS to decrease the Histodenz density, allowing the lysosomes to be pelleted, washed, and resuspended in isotonic MOPS/sucrose buffer. Protein concentrations were measured by BCA assay (ThermoFisher, 23225), and all samples were adjusted to the same protein concentration.

### Lysosome isolation–analysis of endogenous substrate uptake

Leupeptin (dissolved in sterile PBS) was administered by intraperitoneal injection at a dose of 100 mg/kg body weight, in a final volume of approximately 100 μL, as previously described (Zhang et al., 2023; Endicott et al., 2022). Injections were administered between 9 a.m. and 9:30 a.m. for each of the six replicates of the experiment. Mice were dissected between 11 a.m. and 11:30 a.m. All mice were allowed free access to food and water until they were humanely euthanized. Upon dissection, mice were qualitatively assessed for the presence of food in the stomach (and all mice used in the study had food in the stomach).

Freshly dissected liver tissue was immediately washed in ice-cold PBS, until no visible blood remained. All subsequent steps were performed at 4°C. Tissue was diced with scissors and gently dounce homogenized in commercially available fractionation buffers (Thermo; 89839). Nuclei, extracellular matrix, and unbroken cells were removed by 10-min centrifugation at 500 × g. The post-nuclear sample was mixed with iodixanol/OptiPrep to a concentration of 15% OptiPrep and loaded onto a discontinuous 17%–30% OptiPrep (Sigma; D1556) density gradient in ultracentrifuge tubes (Beckman Coulter; 344057) for a 55Ti-SW rotor (Beckman Coulter; 342194). A 10% Optiprep layer was laid over the sample, and the samples were subjected to ultracentrifugation at 145,000 × g for 2 h in a Beckman Coulter L-70 ultracentrifuge. During ultracentrifugation, the sample resolved into visible bands on the density gradient. The visible band at the 10%–15% gradient interface is highly enriched for lysosomal markers and was used for all experiments. After the lysosome-containing band was removed from the density gradient, the sample was diluted in PBS to decrease the OptiPrep density, allowing the lysosomes to be pelleted, washed, and resuspended. Protein concentrations were measured by BCA assay (ThermoFisher; 23225), and all samples were adjusted to the same protein concentration, before being analyzed by Western blotting.

### Western blotting

Western blotting was performed using a standard protocol (Wink et al., 2022; Jiang et al., 2023). Protein gel electrophoresis was performed on 15-well, 4%-20% gradient gels (BioRad: 4561096), with the manufacturer’s recommended Tris/Glycine/SDS running buffer, diluted to 1x (BioRad: 1610732). Proteins were transferred onto PVDF membrane with 0.2 μm pore size (BioRad: 1620177), to maximize the capture of small proteins, such as H3 and LC3. Transfer buffer was Tris/Glycine Buffer (BioRad: 1610734), diluted to 1 x in 80%/20% (v/v) water/methanol (Fisher: A412-4). After the transfer, membranes were rinsed once with water and were placed in blocking buffer 1 x TBS, diluted from 10x stock (BioRad: 1706435) plus 0.1% (v/v) Tween (Sigma: P1379), plus 5% (w/v) dry milk powder (Research Products International: M17200), for 20 min, on a rotating platform. Membranes were cut to appropriate sizes and incubated overnight at 4°C, with rocking, in blocking buffer containing the primary antibodies described above. Membranes were washed once for 5 min in wash buffer (1 × TBS +0.1% (v/v) Tween). HRP-conjugated goat pAb targeting Rabbit IgG secondary antibody (AbCam: ab6721) was diluted in blocking buffer and incubated with membranes for 1 h, on a rotating platform. Membranes were quickly rinsed with water and washed once for 5 min in wash buffer, before being imaged. The HRP substrate was EcoBright Femto (InnovSol: EBFH100). Images were acquired with a GE Healthcare ImageQuant LAS 4000, using the manufacturer’s ImageQuant LAS 4000 software. Files were saved as “.gel” format to preserve all metadata for analysis. Image analysis was performed in FIJI/ImageJ using the “Analyze Gels” function.

### Statistical analysis

All statistical analysis and graph generation were performed with GraphPad Prism 8. Results of t-tests and 2-way ANOVAs are reported directly on the figures or in the figure legends. Unless otherwise stated, all t-tests are two-sided and unpaired.

## Results

### Enzymes for *de novo* lipogenesis (DNL) are reduced by Aca, cana, and CR in mice

**FIGURE 1:**
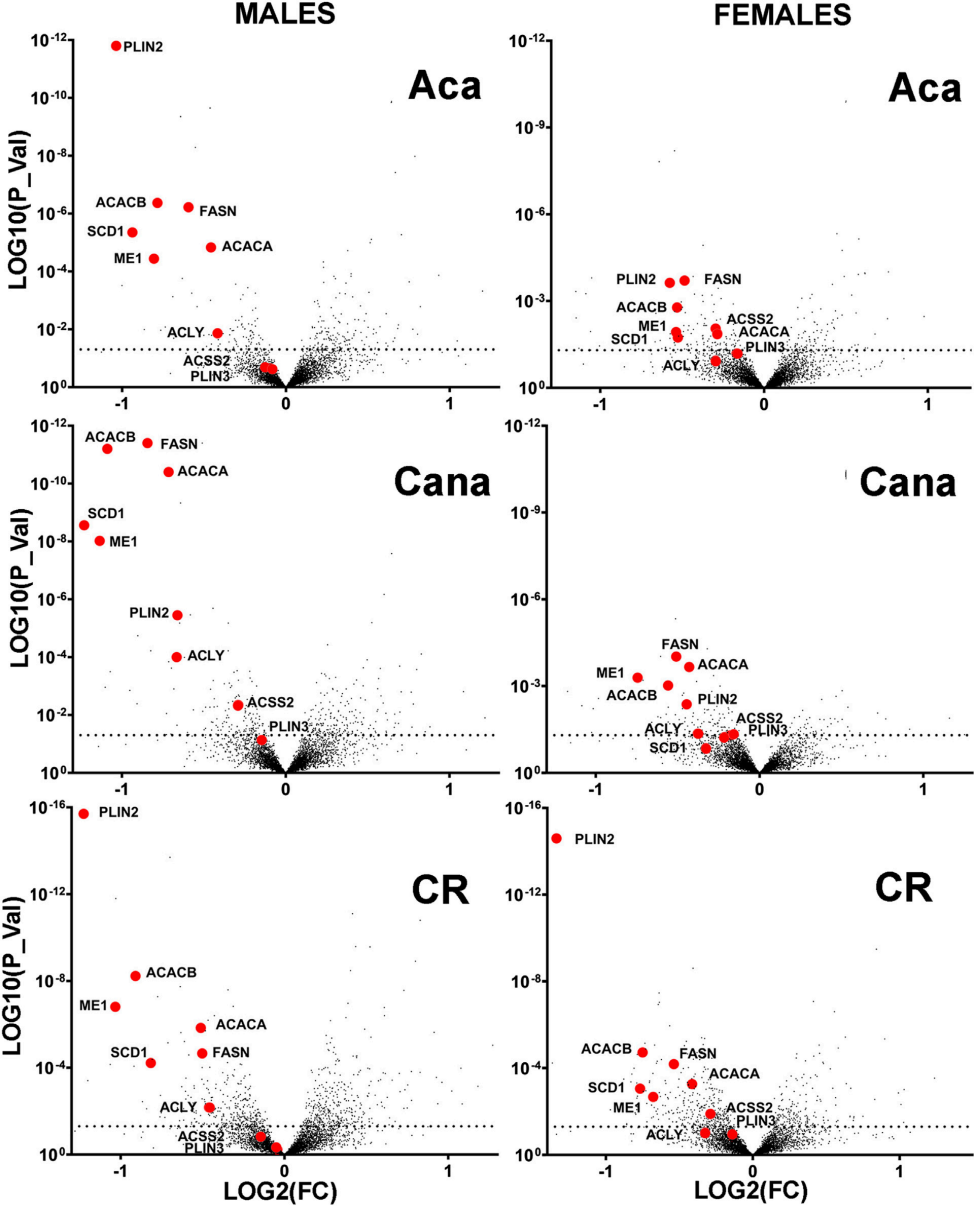
Volcano plots showing intervention-induced change in proteins (Log2 scale, horizontal axis) vs. corresponding p-value (log10 scale, vertical axis) for each of 4293 proteins measured in liver of 12-month-old mice resulting from a minimum of 6 mice per treatment and sex, contrasting to age-matched and sex-matched control mice. The red symbols in each plot indicate a set of nine enzymes from the DNL downregulated in each of the three anti-aging interventions shown.

Our previous work (Endicott et al., 2022) had shown that liver of long-lived mutant mice (Snell dwarf, GHRKO) had reduced levels of several enzymes involved in DNL, because of post-translational changes, that include specifically increases in protein-specific chaperone-mediated autophagy (CMA) as well as declines in eIF4 phosphorylation (Wink et al., 2022; Hsieh and Papaconstantinou, 2004). To see if similar alterations in DNL enzymes were also characteristic of other, non-genetic models of slow aging in mice, we turned to a proteomics dataset (Watanabe et al., 2023; Burns A. R. et al., 2024) which includes liver protein levels from 12-month old mice that had been exposed for 8 months to a calorie-restricted (CR) diet or to Aca or Cana, drugs thought to extend lifespan by reduction in peak blood glucose levels. Figure 1 shows volcano plots for Aca, Cana and CR mice of both sexes. Red symbols indicate 9 enzymes involved in DNL and triglyceride storage. Four of the enzymes involved in *de novo* production of lipids from acetyl-coA, specifically ACACA, FASN, ACLY and ACSS2, had been shown previously to be diminished in liver of Snell and GHRKO mice, possibly through protein-specific degradation via CMA (Endicott et al., 2022). ME1 is an essential component of the citrate-malate shuttle that takes carbon from the mitochondria to supply DNL (Simmen et al., 2020). PLIN2 and PLIN3 can be degraded by CMA to promote the dismantling of lipid droplets and the oxidation of their constituent triglycerides (Kaushik and Cuervo, 2015; Kaushik and Cuervo, 2016). SCD1 is the Acyl-CoA desaturase 1 involved in the regulation of lipid synthesis (Dobrzyn and Ntambi, 2005; Sen et al., 2023). ACACB is a mitochondrial protein involved in acetyl-CoA metabolism and lipid oxidation (O'Neill et al., 2014). A chart of the location and metabolic pathway of these proteins can be found in Supplementary Figure S1.

**FIGURE 2:**
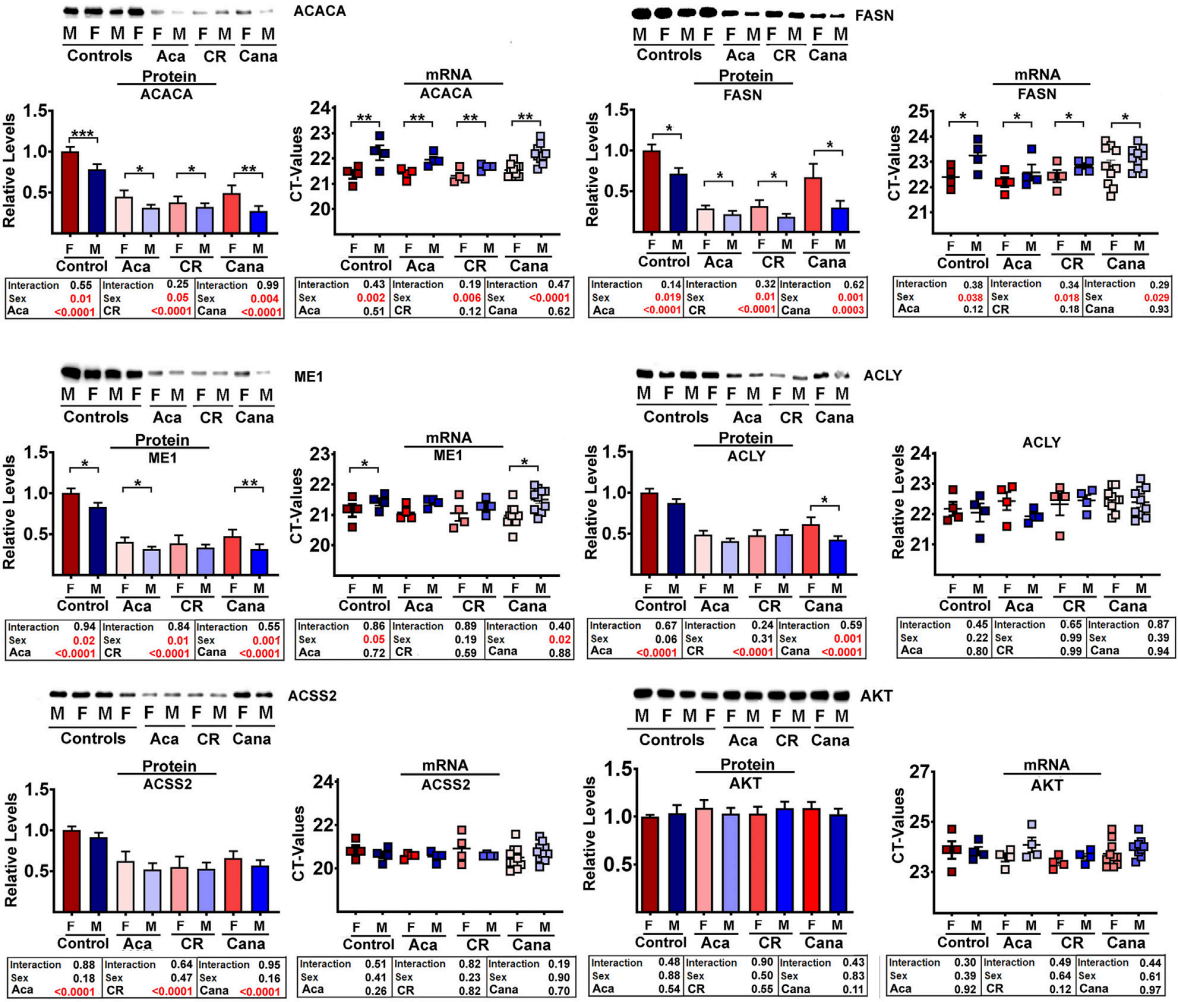
Comparative analysis by Western blots (control n = 9, Aca = 9, Cana = 12, CR = 9 mice per each sex) and qRT-PCR (control n = 4, Aca = 4, Cana = 10, Cr = 4 mice per each sex) of a subset of DNL proteins from liver samples of mice exposed to interventions that extend lifespan; acarbose (Aca), calorie restriction (CR) and canagliflozin (Cana). All interventions show a significant reduction in the set of DNL proteins by western blots (see box below each graph bar). In contrast, the interventions have no effects in the levels of each transcript (see mRNA scatter plot and box below for each protein). AKT was used as internal control for standardization between samples.

The proteomics data showed that all 9 of these enzymes were reduced in liver of each of CR, Aca, and Cana mice, in both sexes, with more dramatic changes typically seen in the male mice. We used immunoblotting to quantitate five of these DNL enzymes in liver of Aca, CR, and Cana mice, to see if we could confirm the results seen in the proteomic survey. Results from ACACA, FASN, ME1, ACLY, and ACSS2 are shown in Figure 2, along with AKT levels as an internal control. In addition, we used RT-PCR to measure mRNA for each of the enzymes from the same samples, and these data are also included in Figure 2. Each of the 5 enzymes showed a significant decline, compared to age- and sex-matched control mice, in each of these three varieties of slow-aging mice. In no case did mRNA levels show an effect of diet or drug treatment, consistent with the hypotheses that the protein levels were controlled by a post-transcriptional process, such as CMA. Each data set was evaluated by two-factor ANOVA. The statistical analysis considered sex, drug or diet, and the interaction term (e.g., Sex x Aca treatment), to determine if the effect of the intervention was dependent on the sex of the mouse. In no case was the p-value for interaction less than 0.05, suggesting that the drugs and CR diet modulated levels of the five enzymes to an equivalent degree in male and female mice, although statistical power may have limited our ability to detect sex-specific effects if these were present.

In addition to the effect of the anti-aging interventions, some of the enzymes showed an effect of sex; specifically, ACACA, FASN, and ME1 were consistently lower in males than in female mice. The mRNA levels for these three proteins were also lower in males, consistent with the idea that the sex effect, unlike the drug and diet effects, may be the effect of sex-specific transcriptional differences or difference in translation between sexes. The control enzyme, AKT, did not show any significant effects of intervention or sex, and did not show any significant [treatment x sex] interactions. Parallel evaluation of protein and mRNA levels for LAMP2A, ERK1, and ERK2 (Supplementary Figure S2A) also showed no effects of intervention or sex, similar to the AKT results.

The proteomic data sets also suggested that enzymes related to DNL were reduced in liver of two varieties of long-lived mutant mice, i.e., GHRKO and Snell Dwarf (see Supplementary Figure S2B) in comparison to levels in their respective littermate control mice. GHRKO mice did not show any alteration of mRNA, suggesting that the effects on these DNL enzymes, like the changes seen in CR, Aca, and Cana mice, reflect post-transcriptional processes. In contrast, however, mRNA levels for these enzymes were downregulated in liver of Snell Dwarf mice. The basis for this discrepancy is unclear, although the endocrine changes in the Snell model include hypothyroidism, which is not characteristic of the GHRKO model.

### Reduction of DNL enzyme levels in mice treated with the ERK1/2 inhibitor trametinib (tram)

**FIGURE 3:**
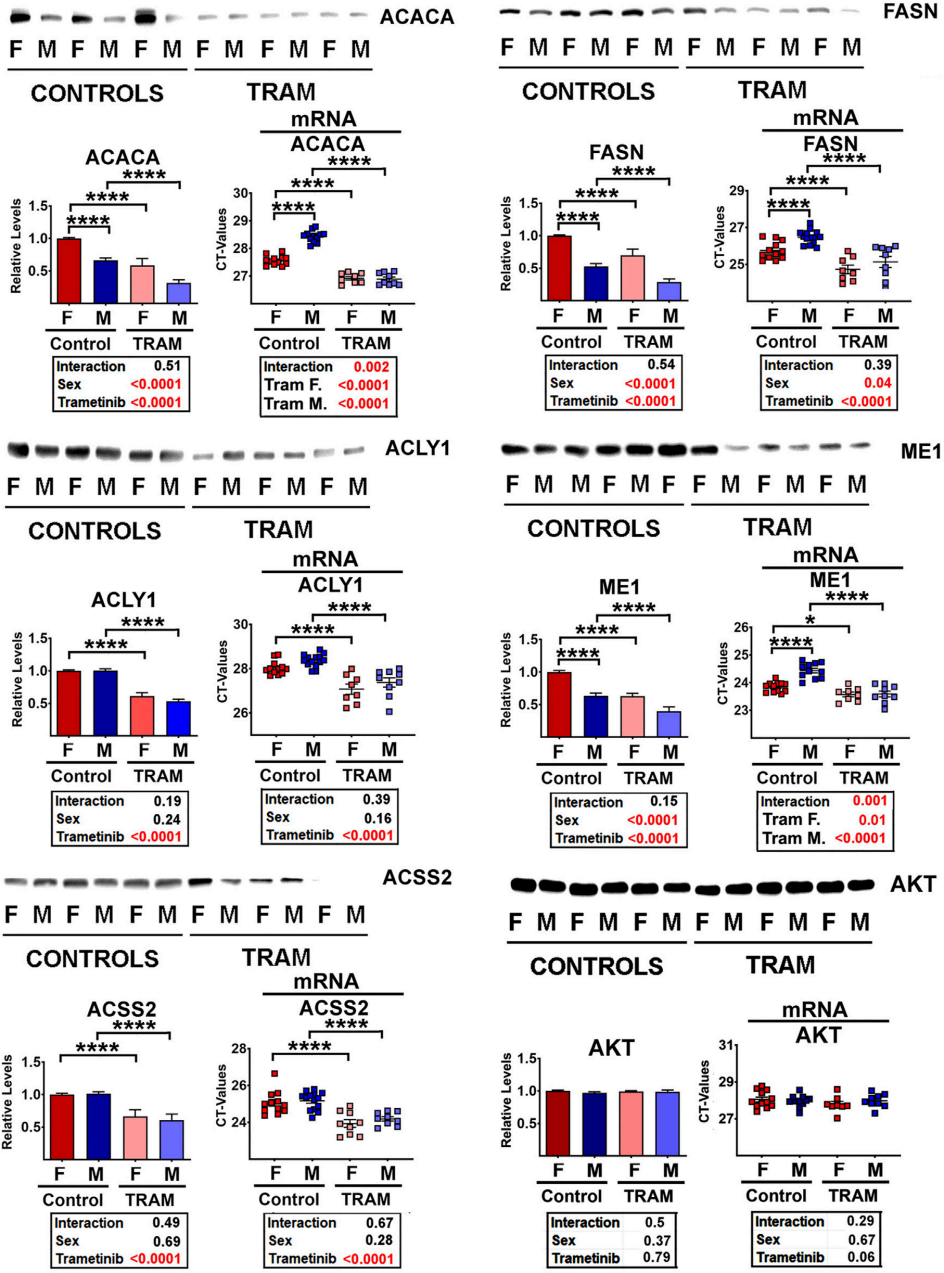
Representative western blots of selected DNL proteins from Tram treated mice, showing mean and SEM from 5 mice per treatment for each sex. Statistical results are shown in the box below each graphic. On the right of each pair of graphics are the data for mRNA for the corresponding proteins, showing (unexpected) increases (i.e., lower CT values) in the levels of each mRNA.

Partridge et al. (Gkioni et al., 2024) have recently shown that trametinib (Tram), an inhibitor of the MEK/ERK kinase pathway, can increase lifespan of both sexes of C3B6F1 mice. Furthermore, when Tram treatment is combined with rapamycin, the two drugs have an additive beneficial effect on mouse lifespan, suggesting that the two drugs have at least some non-overlapping mechanisms for boosting mouse lifespan. To see if Tram would modify enzymes of the DNL pathway, we evaluated liver samples from UM-HET3 mice given this inhibitor for 45 days starting at 23–25 weeks of age. Figure 3 shows that Tram reduces each of the five tested DNL enzymes significantly (p < 0.001 in each case). We also documented significant changes in mRNA levels for each enzyme (Figure 3), but, surprisingly, noted that the drug and diet interventions led to increases in mRNA levels (i.e., lower CT values) despite the decline in protein levels. This disparity in the direction of change between mRNA and proteins suggests that the decline in enzyme levels is, again, mediated by a non-transcriptional pathway, potentially CMA. Sex effects on ACACA, FASN, and ME1 protein levels are noted in Figure 3 as well, with higher DNL enzyme levels in females than in males, consistent with the data from 12-month-old mice shown in Figure 2. The results suggest that some of the effects of Aca, Cana, and CR shown in Figure 2 might reflect downregulation of the MEK/ERK kinase cascade, since they are reproduced by the MEK/ERK inhibitor Tram.

As expected, Tram treated mice showed declines in MEK activity, as indicated by lower levels of phosphorylated ERK1 and ERK2, the key substrates of MEK (Supplementary Figure S3A). Since total amounts of ERK1 and ERK2 are not changed, the decrease in phosphorylated species leads to lower levels of the pERK/ERK ratio, as expected. The phosphorylation of MEK1 itself is increased in liver of Tram-treated mice, possibly as a result of unknown feedback signals. Similarly, Tram leads to lower levels of phosphorylation of substrates of the ERK1/2 kinases, i.e., of MNK1/2 (Supplementary Figure S3A). Phospho-MNK1 cannot be discriminated from phospho-MNK2, and for this reason the graphics are based on overall pMNK. Total amounts of MNK are not altered by Tram treatment, although the data show the sex effect we previously reported (Wink et al., 2022), with higher MNK1 and MNK2 levels in male mice, accompanied by, and presumably caused by, higher levels of the corresponding mRNAs. The activity of MNK, estimated by phosphorylation status of the translation factor eIF4E, was also found to be lowered in Tram-treated mice (Supplementary Figure S3A).

Because inhibition of mTOR is thought to be the key mechanism for rapamycin effects on lifespan (Xu et al., 2014), and because phosphorylation ratios for two mTOR substrates, S6K and 4EBP1, are lower in Aca and Cana mice (Wink et al., 2022; Jiang et al., 2023), we considered the possibility that Tram effects might be mediated, indirectly, by inhibition of mTORC1 action. Supplementary Figure S3B refutes this hypothesis. Neither the ratio of p4EBP1/4EBP1 nor the ratio of pS6/S6 is altered in Tram treated mice. Similarly, there are no changes in phosphorylation ratio of AKT or N-myc downregulated gene 1 (NDRG1), suggesting no Tram effect on function of mTORC2.

In addition, Tram treatment led to a significant decline in body mass in both sexes, with mice losing an average of about 5–6 g (Supplementary Figure S3C). Mass of brown fat, perigonadal fat, and inguinal fat, relative to total body mass, also declined in Tram-treated mice, as did mass of heart and liver suggesting major effects on lipid metabolism and on *de novo* lipogenesis or accumulation in fat tissues. Some of these effects were more prominent in one sex or the other, as shown in the statistical reports included in Supplementary Figure S3D.

### Tram reduces enzymes of DNL in cultured AML12 cells

**FIGURE 4:**
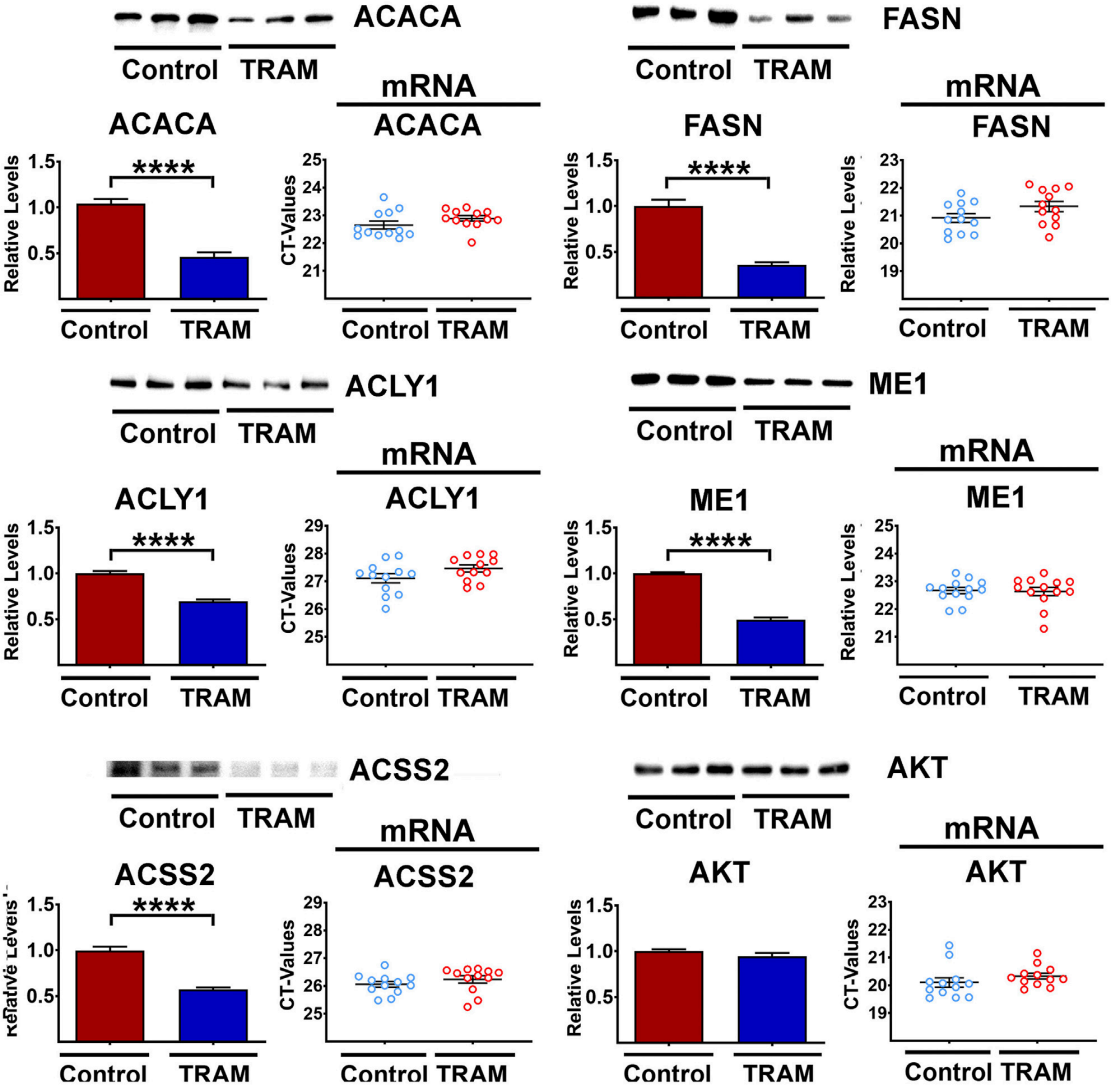
Representative western blots of five DNL proteins in AML cells treated with Tram. The bar graphs show the downregulation (mean ± SEM) of the protein levels. Results reflect 6 independent experiments (totaling 12 data points). The mRNA scatter-plots are shown alongside each of the protein graphics (6 independent experiments (totaling 12 data points)), and show no significant changes in the mRNA levels.

We hypothesized that the effects of Tram on DNL protein levels in the liver might be cell autonomous, rather than a consequence of circulating glucose, insulin, or other endocrine signals altered by Cana, Aca, or CR diet. To test this hypothesis, we turned to an *in vitro* system, in which mouse hepatocyte cells, line AML12, were exposed to Tram at a dose of 25 nM for 48 h and then assessed for DNL enzyme levels. As shown in Figure 4, Tram led to a significant decline in each of the 5 DNL enzymes, without a corresponding change in the mRNAs for any of these enzymes. The disparity between protein and mRNA changes is, again, consistent with post-transcriptional mechanisms. Supplementary Figure S4A confirms that Tram, as expected, diminished the activity of MEK, as indicated by decline in the level of phosphorylation of its substrates ERK1 and ERK2, and decline in the phosphorylation of the substrate of ERK1/2, i.e., MNK. This figure also shows, as expected, decline in the phosphorylation of the MNK substrate eIF4E, a regulation of translation. We speculate that elevated phosphorylation of MEK1, the first MAP kinase in this pathway and also found upregulated *in vivo* with Tram treatments (see Supplementary Figure S3A), may reflect a feedback loop similar to the pathway that elevates AKT phosphorylation in cells exposed to AKT inhibitors (Zhang et al., 2023; Okuzumi et al., 2009).

Because mTORC1 activity is also lower in Aca and Cana mice (Wink et al., 2022; Jiang et al., 2023), we considered the possibility that Tram effects might be mediated, indirectly, by inhibition of mTORC1 action. Supplementary Figure S4B shows that Tram lowers the level of both S6 and 4EBP1 protein, without corresponding change in the level of their mRNAs. The level of the two phosphoproteins declines to the same extent, implying a small but significant decline in mTORC1 kinase action, but the ratio of phosphoprotein to total protein does not change after Tram exposure. It is therefore difficult to draw a clear conclusion as to whether mTORC1 function on AML cells is altered meaningfully. The data in Supplementary Figure S3B, as noted, do not suggest any alterations of mTORC1 function in mice treated with Tram. There are no changes in phosphorylation ratio of AKT or NDRG1 in AML cells, suggesting no Tram effect on function of mTORC2 function in these cultured cells. Parallel work done in liver of Tram-treated mice (the same mice used in Figure 3) found no effects of Tram on mTORC1 or mTORC2 function (Supplementary Figure S4B), and showed the expected decline in activity of MEK1 as indicated by decreased phosphorylation of ERK1, ERK2, MNK, and eIF4E. We did note lower levels of 4EBP1 and S6 not seen in the *in vivo* results (see Supplementary Figure S3C). The basis for this discrepancy is unknown.

### Downregulation of DNL proteins after tram-mediated MEK1 inhibition is the result of CMA activation

**FIGURE 5:**
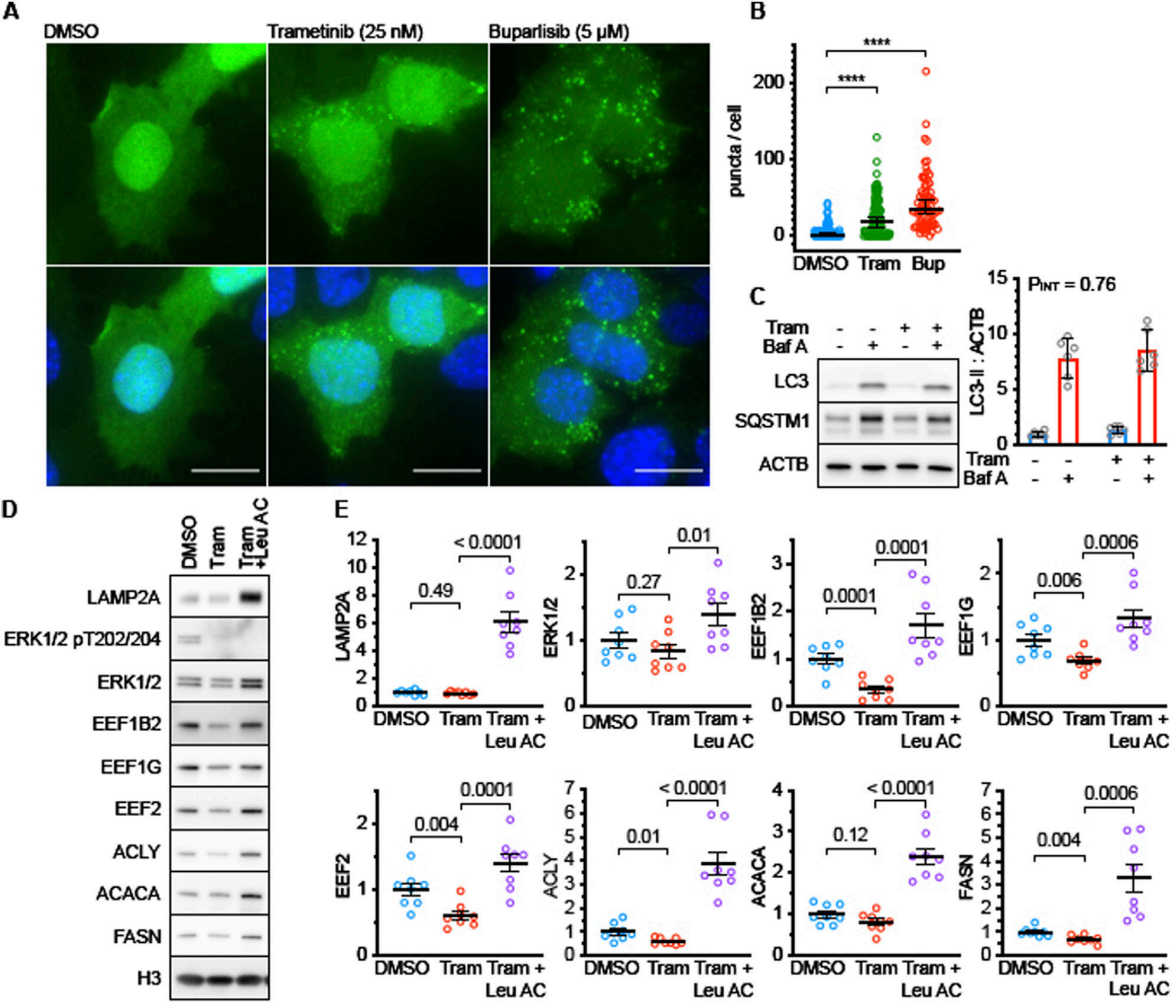
Trametinib enhances CMA activity in AML12 cells. **(A)** Representative fluorescence microscopy images of AML12 cells expressing the KFERQ-Dendra2 CMA reporter, following treatment for 4 h with DMSO (solvent control), Tram, or Buparlisib. Drug concentrations are shown in figure. **(B)** Quantification of results shown in **(A)**. p values represent the results of a Kolmogorov-Smirnov non-parametric test. **(C)** Representative western blots and quantification of a macroautophagic flux assay in AML12 cells treated with Tram for 4 h. n = 6 for each condition. P_INT_ is the interaction term from a 2-factor ANOVA. There is no detectable effect on macroautophagy after 4 h of treatment. **(D)** Representative western blots of lysates from AML12 cell treated for 48 h with Tram or Tram + Leupeptin and Ammonium Chloride (to block lysosomal proteolysis). LAMP2A is a control for effective leupeptin administration. ERK and pERK are controls for effective Tram treatment. EEF1B2, EEF1G, EEF2, ACLY, ACACA, and FASN are CMA substrates. **(E)** Quantifications of blots shown in **(D)**. n = 8 for each condition. P values on the graphs are the results of unpaired t tests, to evaluate if Leupeptin and Ammonium Chloride “rescued” the effects of Trametinib treatment. Levels of histone H3 were measured in each experiment and used to normalize across replicate gels.

Next, we sought to test the hypothesis that changes in protein degradation explained the mRNA-independent reduction of DNL enzymes in Tram treated mice. We have previously shown that DNL enzymes are targets of CMA in the Snell and GHRKO models and that interventions that activate CMA can lead to reductions of these proteins in mouse liver (Zhang et al., 2023; Endicott et al., 2022). To see if ERK pathways were involved in regulation of CMA, we evaluated CMA levels in Tram-treated AML12 cells, using a well-established fluorescent CMA reporter that accumulates on lysosomes when CMA is active (Koga et al., 2011). Tram leads to increases in the mean number of autophagic CMA reporter puncta per cell (Figures 5A,B), with the CMA activator buparlisib (Endicott et al., 2020) used as a positive control. To see if Tram affected macroautophagic flux in AML12 cells, we performed a Bafilomycin A-based LC3 flux assay (Figure 5C). Tram did not modify the effect of Bafilomycin A on sequestosome-1 (SQSTM1) or LC3 (see Figure 5C, p_INT_ = 0.76), implying that it does not affect macroautophagy in this cell line, at least not on the 4-h timescale examined here.

We then tested whether the Tram-induced decline in ACACA, FASN, and ACLY in AML12 cells would be blocked by the combination of leupeptin and ammonium chloride, which inhibit lysosome-mediated degradation of proteins transported by CMA to the interior of the lysosome. As shown in Figures 5D,E, the decline of all three proteins was indeed blocked by leupeptin. Our previous work (Zhang et al., 2023) has shown that three proteins involved in translation elongation, i.e., elongation factor 1-gamma, elongation factor 1 beta-2 and elongation factor 2 are also diminished in liver of long-lived GHRKO mice by means of CMA. We therefore measured levels of all three elongation factors in Tram-exposed AML12 cells with and without leupeptin inhibition of CMA. The results (Figures 5D,E) in each case closely resembled the effects on ACACA, FASN, and ACLY, i.e., a decline in Tram-treated cells that was itself blocked by leupeptin. Tram did not, by itself, alter levels of ERK1/2 or of the lysosome-specific protein LAMP2A (Figures 5D,E), although the levels of both proteins were increased by leupeptin, suggesting regulation of their levels by lysosome-mediated digestion. These data show that the decline in several proteins, including enzymes of DNL and factors related to translation elongation, is mediated by CMA in Tram-treated AML cells.

### Male mice treated with trametinib have increased lysosomal uptake of CMA substrates

To determine if liver lysosomes from Tram treated mice have elevated uptake of endogenous CMA target proteins, we used a well-established lysosomal uptake assay for endogenous proteins (Endicott et al., 2021; Zhang et al., 2023; Endicott et al., 2022). Briefly, mice were treated with Tram or control diets and then were injected with leupeptin, or PBS solvent control, 2 h before euthanasia. Liver lysosomes were then harvested by density centrifugation and tested for uptake of CMA substrates including the DNL enzymes ACACA, FASN, ACLY, and ACSS2. Lysosomal uptake of ENO1 and GAPDH, commonly used as standards for CMA lysosomal uptake assays (Endicott et al., 2021; Zhang et al., 2023; Endicott et al., 2022; Schneider et al., 2014), was included in this protocol.

**FIGURE 6:**
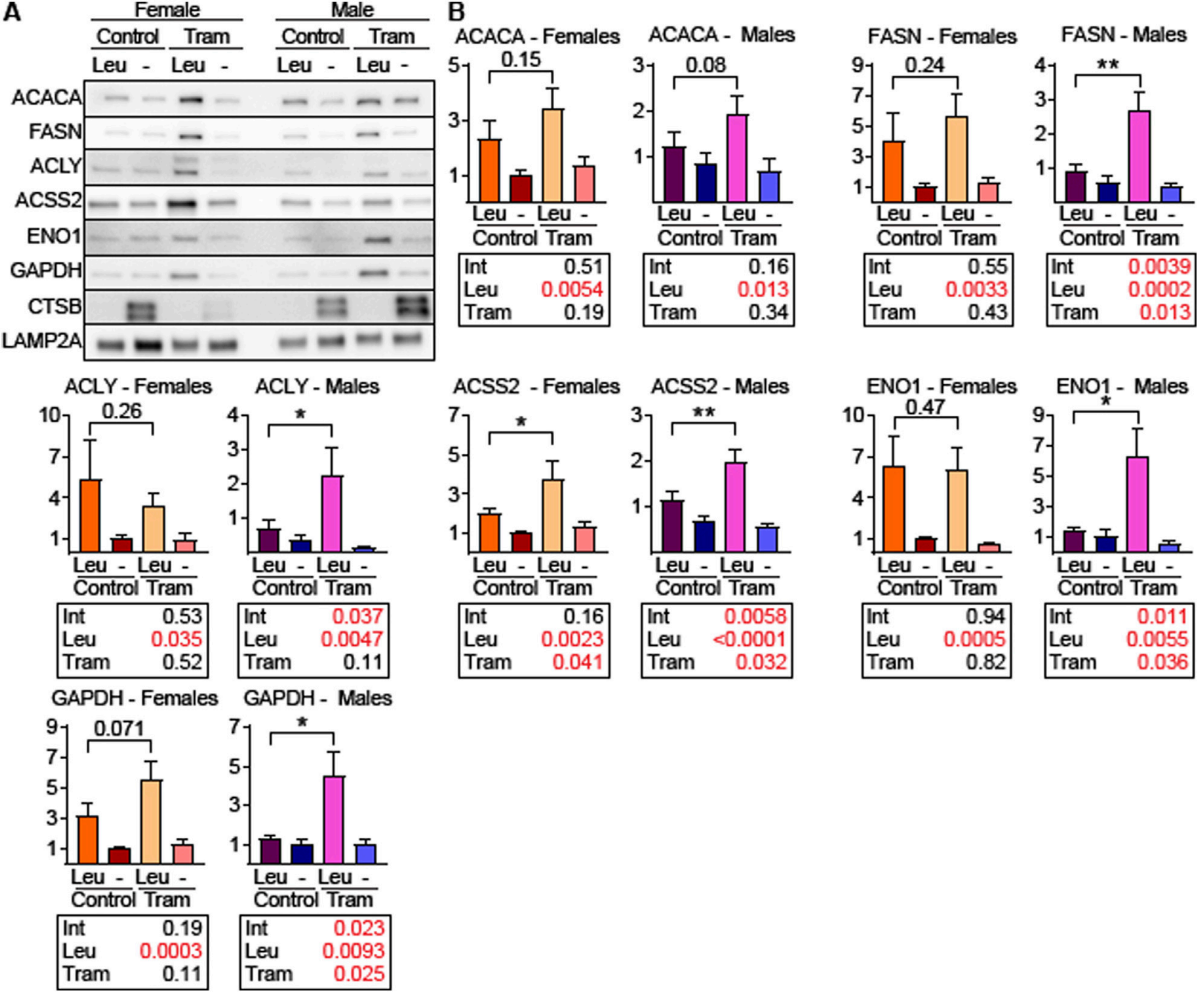
Tram treated male mice have increased uptake of endogenous CMA target proteins into liver lysosomes. **(A)** Representative western blots of the indicated proteins using liver lysosomes from female and male mice on control diet or trametinib diet, isolated from mice following intraperitoneal injection of leupeptin (Leu) or PBS (-). CTSB is a control for the effectiveness of leupeptin injection. LAMP2A is the loading control. **(B)** Quantifications and statistical analysis of blots from **(A)**. Tables below each plot report the results of a 2-factor ANOVA. n = 8 for each condition. Brackets with p values report the results of an unpaired t-test.

Unexpectedly, liver lysosomes from female UMHET3 mice had significantly higher uptake of CMA substrates than lysosomes from males, even on the control diet (Figure 6A). Such dramatic sex differences in CMA have not been reported in other genetic backgrounds (Endicott et al., 2021; Zhang et al., 2023; Endicott et al., 2022). Data for males and females are for this reason plotted separately, and on different scales, in Figure 6B. In this experimental design, the p-value for the [Leu x Tram] interaction term indicates whether the Leupeptin mediated increase in protein uptake (i.e., CMA activity) is itself modified by Tram. We therefore used the Interaction p-value as our criterion for Tram effects on CMA. In male mice, Tram significantly increased the uptake of nearly all tested CMA substrates. The exception was ACACA, which was just outside of statistical significance (p = 0.16; Figure 6B). In female mice, with their elevated baseline level of CMA, Tram did not lead to further activation of CMA, as indicated by the p-value for the Interaction term (Figure 6B).

**FIGURE 7:**
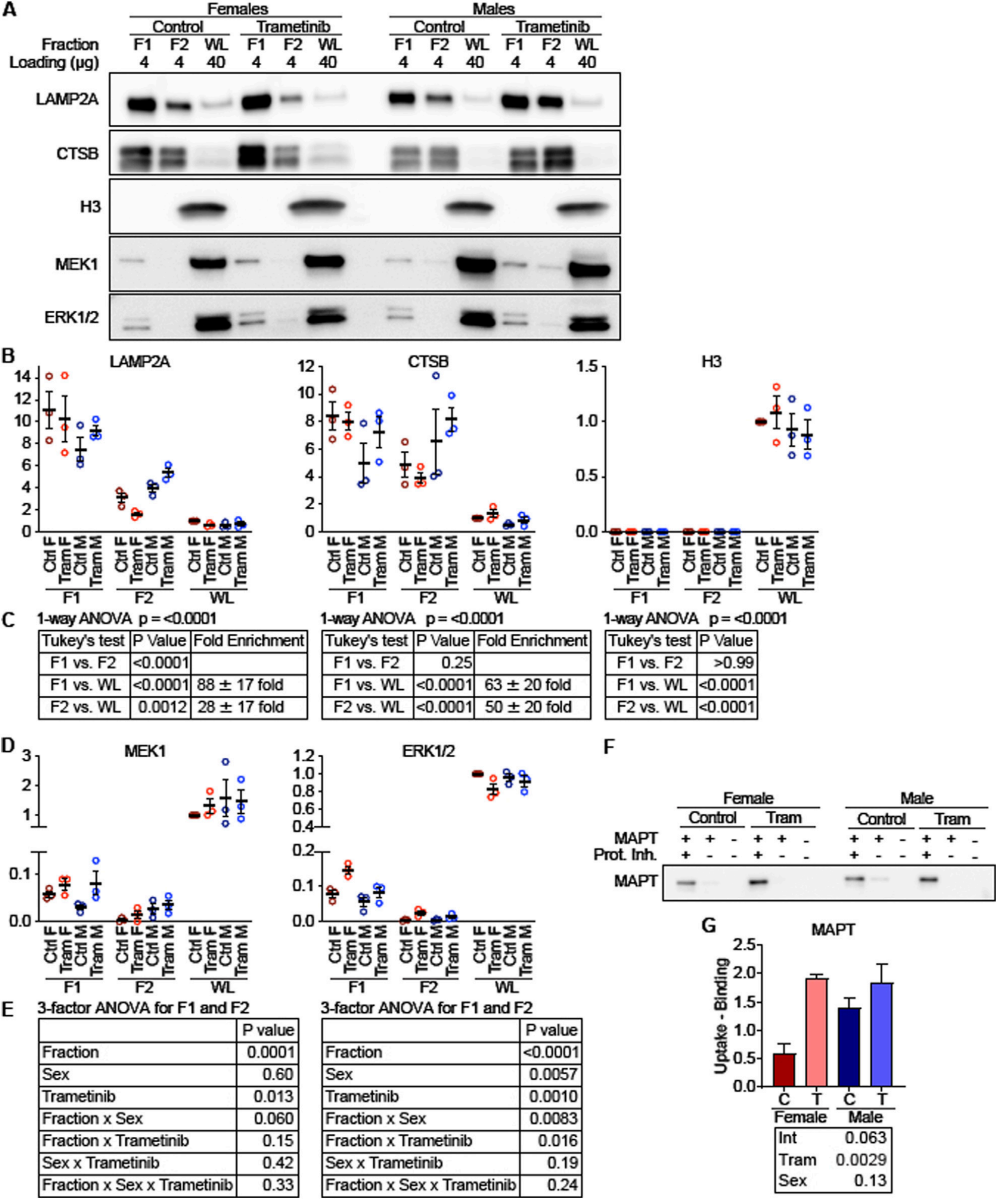
MEK and ERK localize to “CMA + lysosomes” and regulate uptake of a CMA substrate. **(A)** Representative Western blot images depicting low density lysosomes (F1; historically referred to as CMA + lysosomes) and high-density lysosomes (F2; historically referred to as CMA-lysosomes) isolated from mouse livers and whole liver lysates (WL), which were analyzed for lysosomal markers (LAMP2A and CTSB), a nuclear marker (H3), and MEK1 and ERK1/2. The amount of protein loaded is indicated for each lane. **(B)** Plots of the quantified western blots from **(A)** for the indicated proteins, normalized to control female WL. **(C)** To evaluate for enrichment of lysosomal marker, a 1-way ANOVA was performed, pooling all samples for F1, F2, and WL. A Tukey’s *post hoc* test indicates significant enrichment of lysosomal markers in F1 and F2 over the WL fraction, and it indicates a significant de-enrichment of H3 in F1 and F2 over the WL fraction. **(D)** Quantifications of MEK1 and ERK1/2 protein levels, from blots depicted in **(A)**. **(E)** A 3-factor ANOVA comparing values from F1 and F2 reveals that MEK1 is enriched in F1 over F2, but that sex and Tram exert complex effects influencing localization of ERK1/2 to F1 vs. F2. **(F)** Western blot showing A binding and uptake assay was performed in F1 lysosomes, using the well-characterized CMA substrate MAPT. **(G)** Plot of quantified western blots from **(F)**. The statistical table reports the results of a 2-factor ANOVA. N = 3 for all experimental conditions.

As a second method to measure the effects of Tram on CMA, we isolated the light lysosomal subpopulation, F1, which is responsible for most CMA activity, from the livers of mice on the Tram and control diets, using a recently developed method that replaces metrizamide with Histodenz (Burns C. M. et al., 2024). This method produced samples with mean enrichments of LAMP2A of 88-fold for fraction F1 and 28-fold for fraction F2, with no detectable nuclear contamination, estimated by histone H3 levels, as shown by the blot image in Figure 7A and quantitated in Figure 7B, with statistical results shown in Figure 7C. We then performed an *in vitro* binding and uptake assay using recombinant microtubule-associated protein tau (MAPT) – a well-established CMA target protein. As shown in Figure 7F, the light lysosomal fraction F1 from Tram treated mice had higher MAPT uptake (p = 0.0029) compared to F1 lysosomes from mice on the control diet, consistent with the idea that Tram increases uptake of proteins into lysosomes by CMA.

To explore the mechanism through which Tram activates CMA, we evaluated lysosomal subpopulations for the presence of MEK1 and ERK1/2 (Figure 7A). The corresponding dotplots are shown in Figure 7D, and statistical assessments in Figure 7E, using ANOVA with three factors, i.e., Tram treatment, Sex, and lysosomal fraction. For MEK1, the analysis shows significantly higher levels in F1 than in F2, consistent with prior observations of enhanced CMA in the F1 fraction (p < 0.0001). Tram treatment increased the level of MEK1 in both F1 and F2 fractions (p = 0.013). ERK1/2 was also higher in F1 than in F2 lysosomes, similar to the MEK1 distribution. The two-way interaction [Fraction x Trametinib] was significant at p = 0.016 for ERK1/2, showing that Tram effects were different in magnitude between the lysosome fractions, but lysosomes from Tram-treated mice had more ERK1/2 than lysosomes from control mice, regardless of which fraction was considered. Thus, Tram seems to increase the amount of ERK1/2 and MEK1 in the lysosome fraction, F1, which is most active in uptake of CMA substrates.

### Lower phosphorylation of GFAP in lysosomes from tram-treated mice

**FIGURE 8:**
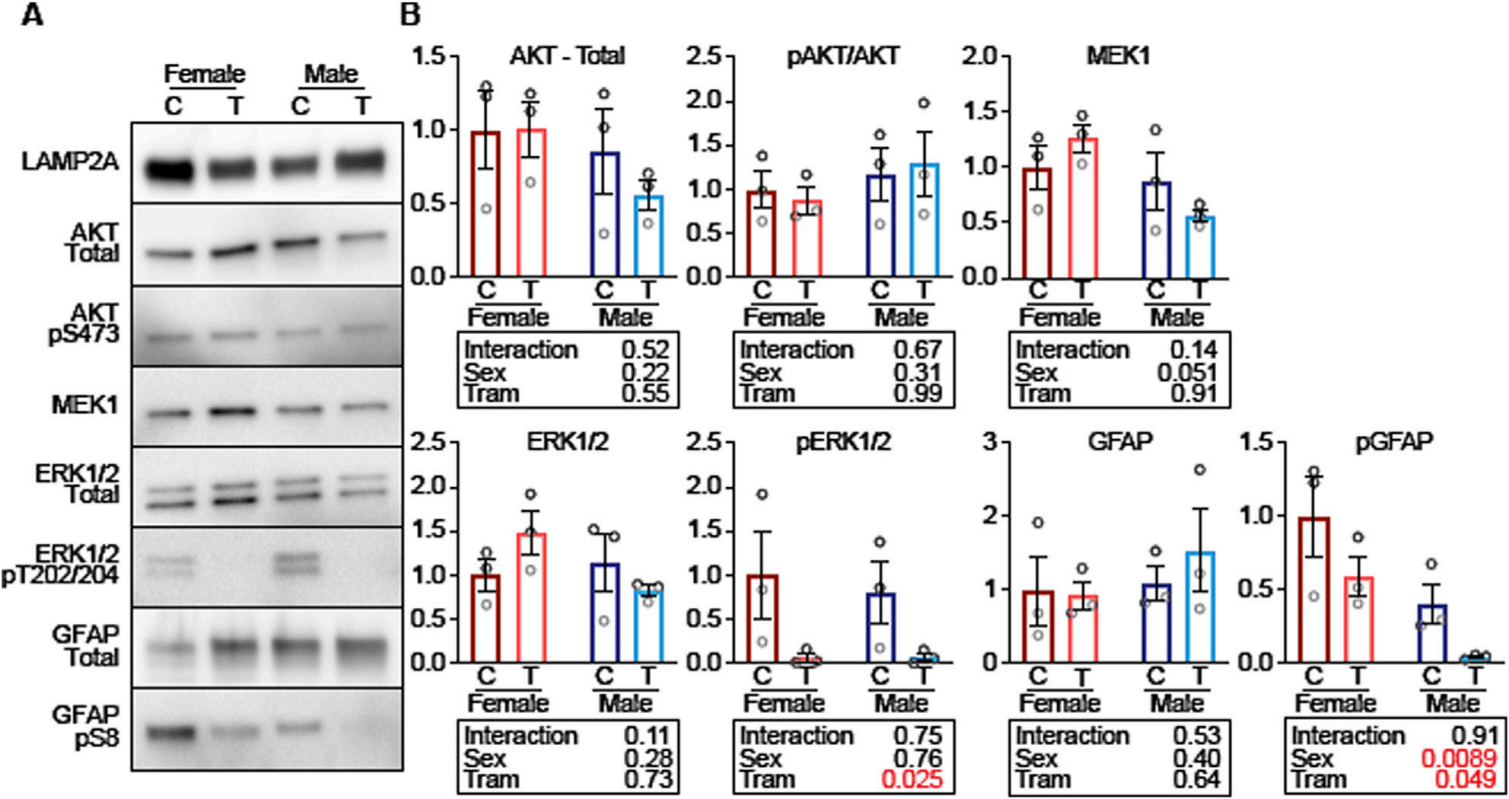
Tram decreases lysosomal GFAP phosphorylation on CMA + mouse liver lysosomes. **(A)** Representative western blots of low-density liver lysosomes from female and male mice on control diet (C) or trametinib diet (T), blotted for the indicated proteins. **(B)** Quantifications and statistical analysis of blots from **(A)**. Tables below each plot report the results of a 2-factor ANOVA. n = 3 for each condition.

F1 fractions from male and female mice, with or without Tram treatment, were then evaluated for their relative levels of proteins involved in CMA activation (Figure 8). The F1 fractions were the same as those used for Figure 7, but the experiment shown in Figure 8 allowed all of the F1 fractions to be evaluated on the same gel, guarding against possible batch effects, and also allowing us to use exposure times appropriate for lysosome fractions rather than for whole cellular lysates. There was no evidence for effects of Tram on either levels of AKT or of pAKT (Figures 8A,B); thus differences in uptake of CMA proteins due to Tram are unlikely to reflect Tram effects on AKT, pAKT, or the pAKT/AKT ratio. Nor does Tram modify the amount of ERK1/2 or MEK1 protein in the F1 lysosomal fraction. The dramatically diminished ratio of pERK/ERK (p = 0.025) reflects the anticipated inhibition of MEK1 function by Tram.

Tram does not lead to a change in the total amount of GFAP in the F1 lysosomal fraction, but does lead to a lower level of pGFAP (p = 0.049). pGFAP levels are higher in F1 lysosomes from females compared to males, but the interaction term is not significant (p = 0.91), showing that Tram-mediated decline in pGFAP is similar in both sexes. Since unphosphorylated GFAP binds to LAMP2A to enhance CMA, but phosphorylated GFAP does not enhance CMA, these data suggest that the decrease in GFAP phosphorylation is a plausible explanation for why CMA is increased in response to trametinib.

## Discussion

Insulin and IGF1, which share overlapping downstream signaling pathways (shown in Supplementary Figure S5), have been implicated in control of the rate of aging in mice. Many varieties of slow-aging, long-lived mice, including mutants such as Snell and Ames dwarf mice, PAPPA-KO mice, and GHR-KO mice, and including mice maintained on a CR diet or to drugs like acarbose and canagliflozin, show a significant reduction in mTOR and MEK-ERK signaling (Wink et al., 2022; Jiang et al., 2023; Dominick et al., 2017; Dominick et al., 2015). These models also show preservation of multiple aspects of health, including diminished sarcopenia, improved glucose homeostasis, and lower inflammation (Fontana, 2018; Masternak and Bartke, 2012; Sadagurski et al., 2015). Less is known, however, about the extent to which these improvements and lifespan and late-life health may depend on shared molecular mechanisms, including alterations in intensity or specificity of mTOR and MEK-ERK signaling, involvement of transcriptional or post-transcriptional pathways, and potential role of sexual dimorphism.

In this paper we have shown that these anti-aging interventions share a common feature: downregulation of hepatic enzymes involved in *de novo* lipogenesis (DNL) (Figures 1, 2). None of the changes in levels of the DNL proteins was accompanied by parallel transcriptional changes (Figure 2; Supplementary Figures S2A–C), strongly suggesting the involvement of post-transcriptional mechanisms. Our data implicate protein-selective CMA as a key post-transcriptional mechanism for these coordinated changes in proteins involved in DNL and imply a role for the MEK1/ERK1/2 kinase pathway inhibited by Tram. It has been shown that reduction of MEK1-ERK signaling can extend the lifespan of mice (Xu et al., 2014) through pathways at least partly independent of mTOR signaling, suggesting a potential signaling pathway regulating DNL levels. Regulation of DNL enzymes by MEK-ERK signals was implied by earlier studies of a knockout mouse lacking MNK1/2 (Sandeman et al., 2020), but the effect was seen only in mice subjected to a high fat diet, and the possible role of non-transcriptional mechanisms was not explored.

To test the idea that the alteration in DNL enzymes might reflect reduction in MEK-ERK signals, we treated young adult UM-HET3 mice with Tram (2.5 ppm in chow for 45 days), and found significant downregulation of DNL levels without corresponding changes at the transcriptional levels, similar to the pattern seen in Snell and GHRKO mice and in mice exposed to Aca, Cana, or CR. The Tram effects on DNL enzymes did not reflect alterations in mTORC1 signals (Supplementary Figure S3C), consistent with models involving post-transcriptional effects on DNL protein levels.

We have previously proposed that elevation of CMA may contribute to extended lifespan of several varieties of slow-aging mice (Endicott and Miller, 2024). Briefly, CMA is negatively regulated by intracellular signaling events downstream of the insulin/IGF1 signaling axis. CMA, in turn, degrades (and reduces the abundance of) proteins such as MYC (Endicott et al., 2021; Gomes et al., 2017), NLRP3 (Yang et al., 2025), ACLY and ACSS2 (Endicott et al., 2022), which are all negative regulators of lifespan. By degrading these negative regulators of lifespan, CMA is responsible for at least some of proteomic and metabolic changes that are known to boost longevity, as reviewed in Endicott (2024). We will discuss the relevant evidence for each of these points, below, and place them in context of the current results.

There are at least four mutations/interventions that extend lifespan in mice, reduce signaling through the insulin/IGF1 cascade, and enhance CMA. These include mutation to pou1f1 (i.e., Snell dwarf), GHRKO (Endicott et al., 2021), PTEN overexpression (Zhang et al., 2023), and calorie restriction (Jafari et al., 2024). Alpelisib, which extends the lifespan of both sexes of C57BL/6 mice (Hedges et al., 2023), is a PIK3CA inhibitor that acts through a similar mechanism of action as known CMA activators buparlisib and pictilisib (Endicott et al., 2020). Additional work will be needed to evaluate the plausible hypothesis that Alpelisib-treated mice have enhanced CMA and associated downstream changes in CMA-modulated proteins. Our current paper adds Tram to the list of lifespan-extending interventions that activate CMA.

The activation of CMA (in mice) by multiple lifespan-extending interventions supports the hypothesis that activation of CMA could promote slowed aging in mice. However, the importance of CMA in healthy aging is further underscored by the consequences of blocking CMA. For example, mice with a liver-specific deletion of essential CMA component, LAMP2A, develop fatty liver disease and a global metabolic dysregulation (Schneider et al., 2014). By 24 months of age, the same mice spontaneously develop liver tumors. Twenty-seven percent of male liver specific LAMP2A KO mice have liver tumors by 24 months of age, with no liver tumors found in controls (Schneider et al., 2015). To some extent, the liver-specific deletion of LAMP2A phenocopies the liver-specific deletion of PTEN. Loss of PTEN reduces CMA in cultured cells (Zhang et al., 2023). Mice with a liver-specific knockout of PTEN have an accumulation of CMA target proteins (Zhang et al., 2023) and suffer global metabolic dysregulation and fatty liver disease that progresses to liver cancer (Stiles et al., 2004). Loss of CMA is also associated with neurodegeneration. For example, mice with a specific deletion of LAMP2A in excitatory neurons are short lived. By 6 months of age, they show declines in neuronal proteostasis, declines in spatial memory, reduced nesting behavior, and an increase in insoluble aggregates of several proteins associated with neurodegeneration (such as SNCA, MAPT, UCHL1, and PARK7) (Bourdenx et al., 2021). Recently generated LAMP2A, LAMP2B, and LAMP2A/B/C knockout models, derived by CRISPR gene targeting, have revealed that LAMP2A and LAMP2B have overlapping roles in macroautophagy in cardiomyocytes (Sampaio Cruz et al., 2025). Future studies using these tools could reveal additional consequences of selective loss of LAMP2A.

Like the other interventions, Tram targets components of signaling cascades downstream of insulin/IGF1. Unlike Alpelisib, however, which inhibits the PI3K/AKT arm of the INS/IGF1 signaling cascade, Tram inhibits the MEK/ERK arm of the cascade. This suggests that both arms are important for modulating CMA activity. The hypothesis that Alpelisib can extend the lifespan of genetically heterogeneous mice has yet to be tested.

Unexpectedly, we found that Tram treatment reduces phosphorylation of lysosomal GFAP. GFAP is an intermediate filament protein that interacts with LAMP2A to promote CMA (Bandyopadhyay et al., 2010). Previously, it was reported that AKT inhibits CMA by phosphorylating lysosomal GFAP and reducing its association with LAMP2A (Arias et al., 2015). However, lysosomal AKT phosphorylation is not affected by Tram. This suggests that the mechanisms that regulate the phosphorylation of lysosomal GFAP might be more complex than originally thought.

In this paper, we have focused on enzymes that produce cytosolic acetyl-coA and enzymes that utilize this pool of acetyl-coA for *de novo* lipogenesis, because they are tightly controlled by CMA in multiple contexts (Zhang et al., 2023; Endicott et al., 2022). Male fruit flies hemizygous for the *Drosophila* ortholog of ACLY show a 32% increase in median lifespan (P et al., 2016). Both male and female flies treated with RNAi targeting the *Drosophila* ortholog of ACSS2 have a significant extension of median lifespan (Eisenberg et al., 2014). It is unclear how rodent lifespan will be affected by genetic alterations that globally reduce ACLY and ACSS2. ACLY and ACSS2 play complex tissue- and cell-type specific roles in metabolism and gene regulation in mammals (Bradshaw, 2021). Thus, tissue-specific knockouts of these genes might be required to fully understand their roles in regulating lifespan. However, the naturally occurring ACLY inhibitor, hydroxycitrate, has properties of a calorie-restriction mimetic, and can reduce adiposity and insulin resistance in obese Zucker rats (Madeo et al., 2019). Male C57BL/6 mice treated with hydroxycitrate in chow showed a significant reduction in early mortality, but no significant change in median lifespan (Espadas et al., 2024). Similarly, inhibition of ACLY with bempedoic acid reduces lipid droplet accumulation and fibrosis in mouse models of diet-induced non-alcoholic fatty liver disease (Morrow et al., 2022). These results correlate, as expected, with results from mice with modified PTEN. Long-lived PTEN overexpressing mice have elevated CMA, and reduced expression of ACLY and ACSS2 (Zhang et al., 2023). PTEN overexpressors do not become obese when fed a high-fat diet, and unlike control siblings do not develop fatty liver disease or develop insulin sensitivity (Ortega-Molina et al., 2012). Reciprocally, PTEN deficiency inhibits CMA (Zhang et al., 2023). Mice with a liver specific deletion of PTEN have increased levels of ACLY and ACSS2 (Zhang et al., 2023), and they develop fatty liver disease by early adulthood (Stiles et al., 2004).

The correlation between mouse lifespan extension and a reduction in ACLY and ACSS2 (and the associated proteins ACACA and FASN) supports the idea that reduction in DNL levels may be an essential mechanism involved in the lifespan of these mice. Furthermore, the reduction in ACLY, ACSS2, ACACA, and FASN are consistently among the most highly downregulated proteins in mice with lifespan extending dietary interventions of Aca, Cana and CR (see Figure 2 and supplemental figures). These interventions all reduce MEK-ERK signaling, and, as shown in Figure 3, inhibition of MEK with Tram is sufficient to reduce the levels of DNL proteins, independent of mRNA changes.

Additional work will be needed to test the hypothesis that Tram reduces the abundance of other CMA target proteins that regulate lifespan, such as MYC and NLRP3. MYC is indirectly controlled by CMA, which degrades CIP2, a protein that indirectly protects MYC from proteasomal degradation (Gomes et al., 2017). Mice that are hemizygous for the MYC gene have a 21% increase in median lifespan for females, with an 11% increase for males (Hofmann et al., 2015). Snell dwarf mice have mRNA-independent reductions in both CIP2A and MYC in multiple tissues (Endicott et al., 2021). Likewise, PTEN overexpressing mice have reduced MYC in several tissue types, although mRNA levels have not been reported (Garc et al., 2012). NLRP3 is a sensor of cellular stressors that, when activated, oligomerizes into an inflammasome, that promotes cellular inflammatory responses (de Torre-Minguela et al., 2017). NLRP3 is negatively regulated by CMA to reduce inflammation (Yang et al., 2025). Two independent studies of NLRP3 knockout mice have found that both sexes have a >30% increase in median lifespan (Navarro-Pando et al., 2021; Marin-Aguilar et al., 2020). Moreover, NLRP3 knockout females have increased litter sizes, and entirely maintain their ability to become pregnant at 12 months of age, at which time, wildtype controls have shown a stark reduction in conception rates (Navarro-Pando et al., 2021). To our knowledge, there have not been any studies that have investigated the hypothesis that CMA downregulates NLRP3 in multiple varieties of long-lived mice.

Because CMA has an important role in regulating the abundance of proteins involved in metabolic dysfunction and age-related diseases, it is highly plausible that selective activation of CMA could promote a healthy metabolism and delay the onset of age-related morbidities. Furthermore, there is a need to test whether known CMA-activating drugs can augment the anti-aging potential of drugs that target other longevity pathways. However, there will be a need to exercise caution in interpreting results from such studies. At present, the pathways known to be involved in CMA regulation, such as the PI3K/AKT (Endicott et al., 2020), MEK/ERK, and RARA pathways (Gomez-Sintes et al., 2022), have broad involvement in several cellular and developmental processes. Thus, there is an important need for future work to identify new interventions that are highly selective and specific for activating CMA.

## Data Availability

The proteomics data presented in this paper were originally published by (Burns et al., 2023), and the source data can be accessed on the PRoteomics IDEntification Database (PRIDE) at https://www.ebi.ac.uk/pride/ using accession number PXD040497.

## Glossary

17aE2: 17 alpha-estradiol
Aca: Acarbose
ACACA: acetyl-CoA carboxylase 1 (cytoplasmic)
ACACB: acetyl-CoA carboxylase 2 (mitochondrial)
ACSS2: acetyl-coenzyme A synthetase (cytoplasmic)
ACLY: ATP-citrate synthase
CIT: cap-independent translation
CMA: chaperone-mediated autophagy
CR: Calorie restriction
Cana: Canagliflozin
DNL: *de novo* lipogenesis
eIF4E: eukaryotic translation initiation factor 4E
4EBP1: eukaryotic translation initiation factor 4E binding protein 1
ERK1/2: extracellular signal-regulated kinase 1 and 2
FASN: fatty acid synthase
GFAP: glial fibrillary acidic protein
GHRKO: growth hormone receptor knockout
HSPA80: Heat shock cognate 71 kDa protein
LAMP2A: lysosome-associated membrane glycoprotein 2
mTORC1: mammalian target of rapamycin complex 1
mTORC2: mammalian target of rapamycin complex 2
ME1: NADP-dependent malic enzyme
MEK1: dual specificity mitogen-activated protein kinase kinase 1
MNK1-2: MAP kinase-interacting serine/threonine-protein kinase 1 and 2
S6: Ribosomal protein S6
Tram: trametinib
PTEN: phosphatidylinositol 3,4,5-trisphosphate 3-phosphatase and dual-specificity protein phosphatase
PLIN1: perilipin-1
PLIN3: perilipin-3
SCD1: acyl-CoA desaturase 1
Snell: Snell Dwarf mice

## References

[B1] Al-Regaiey K. A. Masternak M. M. Bonkowski M. Sun L. Bartke A. (2005). Long-lived growth hormone receptor knockout mice: interaction of reduced insulin-like growth factor i/insulin signaling and caloric restriction. Endocrinology 146, 851–860. 10.1210/en.2004-1120 15498882

[B2] Arias E. Koga H. Diaz A. Mocholi E. Patel B. Cuervo A. M. (2015). Lysosomal mTORC2/PHLPP1/Akt regulate chaperone-mediated autophagy. Mol. Cell 59, 270–284. 10.1016/j.molcel.2015.05.030 26118642 PMC4506737

[B3] Bandyopadhyay U. Sridhar S. Kaushik S. Kiffin R. Cuervo A. M. (2010). Identification of regulators of chaperone-mediated autophagy. Mol. Cell 39, 535–547. 10.1016/j.molcel.2010.08.004 20797626 PMC2945256

[B4] Bourdenx M. Martín-Segura A. Scrivo A. Rodriguez-Navarro J. A. Kaushik S. Tasset I. (2021). Chaperone-mediated autophagy prevents collapse of the neuronal metastable proteome. Cell 184, 2696–2714.e25. 10.1016/j.cell.2021.03.048 33891876 PMC8152331

[B5] Bradshaw P. C. (2021). Acetyl-CoA metabolism and histone acetylation in the regulation of aging and lifespan. Antioxidants (Basel) 10, 572. 10.3390/antiox10040572 33917812 PMC8068152

[B6] Brown-Borg H. M. Borg K. E. Meliska C. J. Bartke A. (1996). Dwarf mice and the ageing process. Nature 384, 33. 10.1038/384033a0 8900272

[B7] Burns A. R. A. R. Wiedrick J. Feryn A. Maes M. Midha M. K. Baxter D. H. (2024). Proteomic changes induced by longevity-promoting interventions in mice. Geroscience 46, 1543–1560. 10.1007/s11357-023-00917-z 37653270 PMC10828338

[B8] Burns C. M. C. M. Miller R. A. Endicott S. J. (2024). Histodenz separation of lysosomal subpopulations for analysis of chaperone-mediated autophagy. Curr. Protoc. 4, e950. 10.1002/cpz1.950 38197533 PMC10874119

[B9] Conn C. S. Yang H. Tom H. J. Ikeda K. Oses-Prieto J. A. Vu H. (2021). The major cap-binding protein eIF4E regulates lipid homeostasis and diet-induced obesity. Nat. Metab. 3, 244–257. 10.1038/s42255-021-00349-z 33619378 PMC10350339

[B10] Conover C. A. Bale L. K. (2007). Loss of pregnancy-associated plasma protein A extends lifespan in mice. Aging Cell 6, 727–729. 10.1111/j.1474-9726.2007.00328.x 17681037

[B11] Conover C. A. Bale L. K. Mader J. R. Mason M. A. Keenan K. P. Marler R. J. (2010). Longevity and age-related pathology of mice deficient in pregnancy-associated plasma protein-A. J. Gerontol. A Biol. Sci. Med. Sci. 65, 590–599. 10.1093/gerona/glq032 20351075 PMC2869530

[B12] Coschigano K. T. Clemmons D. Bellush L. L. Kopchick J. J. (2000). Assessment of growth parameters and life span of GHR/BP gene-disrupted mice. Endocrinology 141, 2608–2613. 10.1210/endo.141.7.7586 10875265

[B13] Cuervo A. M. Dice J. F. (1996). A receptor for the selective uptake and degradation of proteins by lysosomes. Science 273, 501–503. 10.1126/science.273.5274.501 8662539

[B14] Cuervo A. M. Dice J. F. Knecht E. (1997). A population of rat liver lysosomes responsible for the selective uptake and degradation of cytosolic proteins. J. Biol. Chem. 272, 5606–5615. 10.1074/jbc.272.9.5606 9038169

[B15] Debes C. Papadakis A. Grönke S. Karalay Ö. Tain L. S. Mizi A. (2023). Ageing-associated changes in transcriptional elongation influence longevity. Nature 616, 814–821. 10.1038/s41586-023-05922-y 37046086 PMC10132977

[B16] de Torre-Minguela C. Mesa Del Castillo P. Pelegrin P. (2017). The NLRP3 and pyrin inflammasomes: implications in the pathophysiology of autoinflammatory diseases. Front. Immunol. 8, 43. 10.3389/fimmu.2017.00043 28191008 PMC5271383

[B17] Dice J. F. (1990). Peptide sequences that target cytosolic proteins for lysosomal proteolysis. Trends Biochem. Sci. 15, 305–309. 10.1016/0968-0004(90)90019-8 2204156

[B18] Dobrzyn A. Ntambi J. M. (2005). The role of stearoyl-CoA desaturase in the control of metabolism. Prostagl. Leukot. Essent. Fat. Acids 73, 35–41. 10.1016/j.plefa.2005.04.011 15941655

[B19] Dominick G. Berryman D. E. List E. O. Kopchick J. J. Li X. Miller R. A. (2015). Regulation of mTOR activity in Snell dwarf and GH receptor gene-disrupted mice. Endocrinology 156, 565–575. 10.1210/en.2014-1690 25456069 PMC4298324

[B20] Dominick G. Bowman J. Li X. Miller R. A. Garcia G. G. (2017). mTOR regulates the expression of DNA damage response enzymes in long-lived Snell dwarf, GHRKO, and PAPPA-KO mice. Aging Cell 16, 52–60. 10.1111/acel.12525 27618784 PMC5242303

[B21] Duan Y. Evans D. S. Miller R. A. Schork N. J. Cummings S. R. Girke T. (2020). signatureSearch: environment for gene expression signature searching and functional interpretation. Nucleic Acids Res. 48, e124. 10.1093/nar/gkaa878 33068417 PMC7708038

[B22] Eisenberg T. Schroeder S. Andryushkova A. Pendl T. Küttner V. Bhukel A. (2014). Nucleocytosolic depletion of the energy metabolite acetyl-coenzyme a stimulates autophagy and prolongs lifespan. Cell Metab. 19, 431–444. 10.1016/j.cmet.2014.02.010 24606900 PMC3988959

[B23] Endicott S. J. (2024). Chaperone-mediated autophagy as a modulator of aging and longevity. Front. Aging 5, 1509400. 10.3389/fragi.2024.1509400 39687864 PMC11647017

[B24] Endicott S. J. Boynton D. N. Jr. Beckmann L. J. Miller R. A. (2021). Long-lived mice with reduced growth hormone signaling have a constitutive upregulation of hepatic chaperone-mediated autophagy. Autophagy 17, 612–625. 10.1080/15548627.2020.1725378 32013718 PMC8032237

[B25] Endicott S. J. Miller R. A. (2024). PTEN activates chaperone-mediated autophagy to regulate metabolism. Autophagy 20, 216–217. 10.1080/15548627.2023.2255966 37669771 PMC10761071

[B26] Endicott S. J. Monovich A. C. Huang E. L. Henry E. I. Boynton D. N. Beckmann L. J. (2022). Lysosomal targetomics of ghr KO mice shows chaperone-mediated autophagy degrades nucleocytosolic acetyl-coA enzymes. Autophagy 18, 1551–1571. 10.1080/15548627.2021.1990670 34704522 PMC9298451

[B27] Endicott S. J. Ziemba Z. J. Beckmann L. J. Boynton D. N. Miller R. A. (2020). Inhibition of class I PI3K enhances chaperone-mediated autophagy. J. Cell Biol. 219, e202001031. 10.1083/jcb.202001031 33048163 PMC7557678

[B28] Espadas I. Cáliz-Molina M. Á. López-Fernández-Sobrino R. Panadero-Morón C. Sola-García A. Soriano-Navarro M. (2024). Hydroxycitrate delays early mortality in mice and promotes muscle regeneration while inducing a rich hepatic energetic status. Aging Cell 23, e14205. 10.1111/acel.14205 38760909 PMC11488303

[B29] Flurkey K. Papaconstantinou J. Miller R. A. Harrison D. E. (2001). Lifespan extension and delayed immune and collagen aging in mutant mice with defects in growth hormone production. Proc. Natl. Acad. Sci. U. S. A. 98, 6736–6741. 10.1073/pnas.111158898 11371619 PMC34422

[B30] Fontana L. (2018). Interventions to promote cardiometabolic health and slow cardiovascular ageing. Nat. Rev. Cardiol. 15, 566–577. 10.1038/s41569-018-0026-8 29795242

[B31] Garcia-Cao I. Song M. S. Hobbs R. M. Laurent G. Giorgi C. de Boer V. C. J. (2012). Systemic elevation of PTEN induces a tumor-suppressive metabolic state. Cell 149, 49–62. 10.1016/j.cell.2012.02.030 22401813 PMC3319228

[B32] Gkioni L. Nespital T. Monzó C. Bali J. Nassr T. Cremer A. L. (2024). A combination of the geroprotectors trametinib and rapamycin is more effective than either drug alone. bioRxiv. 10.1101/2024.07.25.605097 PMC12270913 40437307

[B33] Gomes L. R. Menck C. F. M. Cuervo A. M. (2017). Chaperone-mediated autophagy prevents cellular transformation by regulating MYC proteasomal degradation. Autophagy 13, 928–940. 10.1080/15548627.2017.1293767 28410006 PMC5446085

[B34] Gomez-Sintes R. Xin Q. Jimenez-Loygorri J. I. McCabe M. Diaz A. Garner T. P. (2022). Targeting retinoic acid receptor alpha-corepressor interaction activates chaperone-mediated autophagy and protects against retinal degeneration. Nat. Commun. 13, 4220. 10.1038/s41467-022-31869-1 35864098 PMC9304322

[B35] Gueugneau M. Coudy-Gandilhon C. Chambon C. Verney J. Taillandier D. Combaret L. (2021). Muscle proteomic and transcriptomic profiling of healthy aging and metabolic syndrome in men. Int. J. Mol. Sci. 22, 4205. 10.3390/ijms22084205 33921590 PMC8074053

[B36] Han Y. Li L. Z. Kastury N. L. Thomas C. T. Lam M. P. Y. Lau E. (2021). Transcriptome features of striated muscle aging and predictability of protein level changes. Mol. Omics 17, 796–808. 10.1039/d1mo00178g 34328155 PMC8511094

[B37] Harrison D. E. Strong R. Allison D. B. Ames B. N. Astle C. M. Atamna H. (2014). Acarbose, 17-alpha-estradiol, and nordihydroguaiaretic acid extend mouse lifespan preferentially in males. Aging Cell 13, 273–282. 10.1111/acel.12170 24245565 PMC3954939

[B38] Harrison D. E. Strong R. Reifsnyder P. Rosenthal N. Korstanje R. Fernandez E. (2023). Astaxanthin and meclizine extend lifespan in UM-HET3 male mice; fisetin, SG1002 (hydrogen sulfide donor), dimethyl fumarate, mycophenolic acid, and 4-phenylbutyrate do not significantly affect lifespan in either sex at the doses and schedules used. Geroscience 46, 795–816. 10.1007/s11357-023-01011-0 38041783 PMC10828146

[B39] Harrison D. E. Strong R. Sharp Z. D. Nelson J. F. Astle C. M. Flurkey K. (2009). Rapamycin fed late in life extends lifespan in genetically heterogeneous mice. Nature 460, 392–395. 10.1038/nature08221 19587680 PMC2786175

[B40] Hedges C. P. Shetty B. Broome S. C. MacRae C. Koutsifeli P. Buckels E. J. (2023). Dietary supplementation of clinically utilized PI3K p110α inhibitor extends the lifespan of male and female mice. Nat. Aging 3, 162–172. 10.1038/s43587-022-00349-y 37118113

[B41] Hofmann J. W. Zhao X. De Cecco M. Peterson A. L. Pagliaroli L. Manivannan J. (2015). Reduced expression of MYC increases longevity and enhances healthspan. Cell 160, 477–488. 10.1016/j.cell.2014.12.016 25619689 PMC4624921

[B42] Horowitz A. M. Fan X. Bieri G. Smith L. K. Sanchez-Diaz C. I. Schroer A. B. (2020). Blood factors transfer beneficial effects of exercise on neurogenesis and cognition to the aged brain. Science 369, 167–173. 10.1126/science.aaw2622 32646997 PMC7879650

[B43] Hsieh C. C. Papaconstantinou J. (2004). Akt/PKB and p38 MAPK signaling, translational initiation and longevity in Snell dwarf mouse livers. Mech. Ageing Dev. 125, 785–798. 10.1016/j.mad.2004.07.008 15541773

[B44] Jafari M. Macho-González A. Diaz A. Lindenau K. Santiago-Fernández O. Zeng M. (2024). Calorie restriction and calorie-restriction mimetics activate chaperone-mediated autophagy. Proc. Natl. Acad. Sci. U. S. A. 121, e2317945121. 10.1073/pnas.2317945121 38889154 PMC11214046

[B45] Jiang E. Dinesh A. Jadhav S. Miller R. A. Garcia G. G. (2023). Canagliflozin shares common mTOR and MAPK signaling mechanisms with other lifespan extension treatments. Life Sci. 328, 121904. 10.1016/j.lfs.2023.121904 37406767 PMC11351721

[B46] Kaushik S. Cuervo A. M. (2015). Degradation of lipid droplet-associated proteins by chaperone-mediated autophagy facilitates lipolysis. Nat. Cell Biol. 17, 759–770. 10.1038/ncb3166 25961502 PMC4449813

[B47] Kaushik S. Cuervo A. M. (2016). AMPK-dependent phosphorylation of lipid droplet protein PLIN2 triggers its degradation by CMA. Autophagy 12, 432–438. 10.1080/15548627.2015.1124226 26902588 PMC4835968

[B48] Kaushik S. Cuervo A. M. (2018). The coming of age of chaperone-mediated autophagy. Nat. Rev. Mol. Cell Biol. 19, 365–381. 10.1038/s41580-018-0001-6 29626215 PMC6399518

[B49] Koga H. Martinez-Vicente M. Macian F. Verkhusha V. V. Cuervo A. M. (2011). A photoconvertible fluorescent reporter to track chaperone-mediated autophagy. Nat. Commun. 2, 386. 10.1038/ncomms1393 21750540 PMC3529934

[B50] Leppek K. Das R. Barna M. (2018). Functional 5' UTR mRNA structures in eukaryotic translation regulation and how to find them. Nat. Rev. Mol. Cell Biol. 19, 158–174. 10.1038/nrm.2017.103 29165424 PMC5820134

[B51] Li X. Shi X. McPherson M. Hager M. Garcia G. G. Miller R. A. (2022). Cap-independent translation of GPLD1 enhances markers of brain health in long-lived mutant and drug-treated mice. Aging Cell 21, e13685. 10.1111/acel.13685 35930768 PMC9470888

[B52] Liu W. Zhu P. Li M. Li Z. Yu Y. Liu G. (2023). Large-scale across species transcriptomic analysis identifies genetic selection signatures associated with longevity in mammals. EMBO J. 42, e112740. 10.15252/embj.2022112740 37427458 PMC10476176

[B53] Madeo F. Carmona-Gutierrez D. Hofer S. J. Kroemer G. (2019). Caloric restriction mimetics against age-associated disease: targets, mechanisms, and therapeutic potential. Cell Metab. 29, 592–610. 10.1016/j.cmet.2019.01.018 30840912

[B54] Maeda H. Gleiser C. A. Masoro E. J. Murata I. McMahan C. A. Yu B. P. (1985). Nutritional influences on aging of Fischer 344 rats: II. Pathology. J. Gerontol. 671–688. 10.1093/geronj/40.6.657 4056322

[B55] Mahalingam M. Cooper J. A. (2001). Phosphorylation of mammalian eIF4E by Mnk1 and Mnk2: tantalizing prospects for a role in translation. Prog. Mol. Subcell. Biol. 27, 132–142. 11575158

[B56] Marin-Aguilar F. Lechuga-Vieco A. V. Alcocer-Gómez E. Castejón-Vega B. Lucas J. Garrido C. (2020). NLRP3 inflammasome suppression improves longevity and prevents cardiac aging in male mice. Aging Cell 19, e13050. 10.1111/acel.13050 31625260 PMC6974709

[B57] Masoro E. J. Yu B. P. Bertrand H. A. (1982). Action of food restriction in delaying the aging process. Proc. Natl. Acad. Sci. U. S. A. 79, 4239–4241. 10.1073/pnas.79.13.4239 6955798 PMC346614

[B58] Masternak M. M. Bartke A. (2012). Growth hormone, inflammation and aging. Pathobiol. Aging Age Relat. Dis. 2, 17293. 10.3402/pba.v2i0.17293 PMC3417471 22953033

[B59] Merrick W. C. (2004). Cap-dependent and cap-independent translation in eukaryotic systems. Gene 332, 1–11. 10.1016/j.gene.2004.02.051 15145049

[B60] Miller R. A. Buehner G. Chang Y. Harper J. M. Sigler R. Smith-Wheelock M. (2005). Methionine-deficient diet extends mouse lifespan, slows immune and lens aging, alters glucose, T4, IGF-I and insulin levels, and increases hepatocyte MIF levels and stress resistance. Aging Cell 4, 119–125. 10.1111/j.1474-9726.2005.00152.x 15924568 PMC7159399

[B61] Miller R. A. Harrison D. E. Allison D. B. Bogue M. Debarba L. Diaz V. (2020). Canagliflozin extends life span in genetically heterogeneous male but not female mice. JCI Insight 5, e140019. 10.1172/jci.insight.140019 32990681 PMC7710304

[B62] Miller R. A. Harrison D. E. Cortopassi G. A. Dehghan I. Fernandez E. Garratt M. (2024). Lifespan effects in male UM-HET3 mice treated with sodium thiosulfate, 16-hydroxyestriol, and late-start canagliflozin. Geroscience 46, 4657–4670. 10.1007/s11357-024-01176-2 38753230 PMC11336000

[B63] Miller R. A. Li X. Garcia G. (2023). Aging rate indicators: speedometers for aging research in mice. Aging Biol. 1, 20230003. 10.59368/agingbio.20230003 PMC10486275 37694163

[B64] Morrow M. R. Batchuluun B. Wu J. Ahmadi E. Leroux J. M. Mohammadi-Shemirani P. (2022). Inhibition of ATP-citrate lyase improves NASH, liver fibrosis, and dyslipidemia. Cell Metab. 34, 919–936.e8. 10.1016/j.cmet.2022.05.004 35675800

[B65] Navarro-Pando J. M. Alcocer-Gómez E. Castejón-Vega B. Navarro-Villarán E. Condés-Hervás M. Mundi-Roldan M. (2021). Inhibition of the NLRP3 inflammasome prevents ovarian aging. Sci. Adv. 7, eabc7409. 10.1126/sciadv.abc7409 33523841 PMC7775749

[B66] Okuzumi T. Fiedler D. Zhang C. Gray D. C. Aizenstein B. Hoffman R. (2009). Inhibitor hijacking of Akt activation. Nat. Chem. Biol. 5, 484–493. 10.1038/nchembio.183 19465931 PMC2783590

[B67] O'Neill H. M. Lally J. S. Galic S. Thomas M. Azizi P. D. Fullerton M. D. (2014). AMPK phosphorylation of ACC2 is required for skeletal muscle fatty acid oxidation and insulin sensitivity in mice. Diabetologia 57, 1693–1702. 10.1007/s00125-014-3273-1 24913514

[B68] Ori A. Toyama B. H. Harris M. S. Bock T. Iskar M. Bork P. (2015). Integrated transcriptome and proteome analyses reveal organ-specific proteome deterioration in old rats. Cell Syst. 1, 224–237. 10.1016/j.cels.2015.08.012 27135913 PMC4802414

[B69] Ortega-Molina A. Efeyan A. Lopez-Guadamillas E. Muñoz-Martin M. Gómez-López G. Cañamero M. (2012). Pten positively regulates brown adipose function, energy expenditure, and longevity. Cell Metab. 15, 382–394. 10.1016/j.cmet.2012.02.001 22405073

[B70] Ozkurede U. Kala R. Johnson C. Shen Z. Miller R. A. Garcia G. G. (2019). Cap-independent mRNA translation is upregulated in long-lived endocrine mutant mice. J. Mol. Endocrinol. 63, 123–138. 10.1530/JME-19-0021 31357177 PMC6691957

[B71] Papaconstantinou J. Deford J. H. Gerstner A. Hsieh C. C. Boylston W. H. Guigneaux M. M. (2005). Hepatic gene and protein expression of primary components of the IGF-I axis in long lived Snell dwarf mice. Mech. Ageing Dev. 126, 692–704. 10.1016/j.mad.2005.01.002 15888324

[B72] Peleg S. Feller C. Forne I. Schiller E. Sévin D. C. Schauer T. (2016). Life span extension by targeting a link between metabolism and histone acetylation in Drosophila. EMBO Rep. 17, 455–469. 10.15252/embr.201541132 26781291 PMC4772992

[B73] Sadagurski M. Landeryou T. Cady G. Kopchick J. J. List E. O. Berryman D. E. (2015). Growth hormone modulates hypothalamic inflammation in long-lived pituitary dwarf mice. Aging Cell 14, 1045–1054. 10.1111/acel.12382 26268661 PMC4693470

[B74] Sampaio Cruz M. Manso A. M. Soto-Hermida A. Bushway P. Silver E. Gunes B. B. (2025). Overlapping functions between Lamp2a and Lamp2b in cardiac autophagy. Autophagy, 1–12. 10.1080/15548627.2025.2484620 PMC12366817 40202173

[B75] Sandeman L. Y. Kang W. X. Wang X. Jensen K. B. Wong D. (2020). Disabling MNK protein kinases promotes oxidative metabolism and protects against diet-induced obesity. Mol. Metab. 42, 101054. 10.1016/j.molmet.2020.101054 32712434 PMC7476876

[B76] Schneider J. L. Suh Y. Cuervo A. M. (2014). Deficient chaperone-mediated autophagy in liver leads to metabolic dysregulation. Cell Metab. 20, 417–432. 10.1016/j.cmet.2014.06.009 25043815 PMC4156578

[B77] Schneider J. L. Villarroya J. Diaz-Carretero A. Patel B. Urbanska A. M. Thi M. M. (2015). Loss of hepatic chaperone-mediated autophagy accelerates proteostasis failure in aging. Aging Cell 14, 249–264. 10.1111/acel.12310 25620427 PMC4364837

[B78] Sen U. Coleman C. Sen T. (2023). Stearoyl coenzyme A desaturase-1: multitasker in cancer, metabolism, and ferroptosis. Trends Cancer 9, 480–489. 10.1016/j.trecan.2023.03.003 37029018

[B79] Shen Z. Hinson A. Miller R. A. Garcia G. G. (2021). Cap-independent translation: a shared mechanism for lifespan extension by rapamycin, acarbose, and 17α-estradiol. Aging Cell 20, e13345. 10.1111/acel.13345 33742521 PMC8135077

[B80] Simmen F. A. Alhallak I. Simmen R. C. M. (2020). Malic enzyme 1 (ME1) in the biology of cancer: it is not just intermediary metabolism. J. Mol. Endocrinol. 65, R77–R90. 10.1530/JME-20-0176 33064660 PMC7577320

[B81] Snyder J. M. Casey K. M. Galecki A. Harrison D. E. Jayarathne H. Kumar N. (2022). Canagliflozin retards age-related lesions in heart, kidney, liver, and adrenal gland in genetically heterogenous male mice. Geroscience 45, 385–397. 10.1007/s11357-022-00641-0 35974129 PMC9886729

[B82] Solyga M. Majumdar A. Besse F. (2024). Regulating translation in aging: from global to gene-specific mechanisms. EMBO Rep. 25, 5265–5276. 10.1038/s44319-024-00315-2 39562712 PMC11624266

[B83] Stiles B. Wang Y. Stahl A. Bassilian S. Lee W. P. Kim Y. J. (2004). Liver-specific deletion of negative regulator Pten results in fatty liver and insulin hypersensitivity [corrected]. Proc. Natl. Acad. Sci. U. S. A. 101, 2082–2087. 10.1073/pnas.0308617100 14769918 PMC357055

[B84] Takemon Y. Chick J. M. Gerdes Gyuricza I. Skelly D. A. Devuyst O. Gygi S. P. (2021). Proteomic and transcriptomic profiling reveal different aspects of aging in the kidney. eLife 10, e62585. 10.7554/eLife.62585 33687326 PMC8096428

[B85] Tyshkovskiy A. Bozaykut P. Borodinova A. A. Gerashchenko M. V. Ables G. P. Garratt M. (2019). Identification and application of gene expression signatures associated with lifespan extension. Cell Metab. 30, 573–593.e8. 10.1016/j.cmet.2019.06.018 31353263 PMC6907080

[B86] Tyshkovskiy A. Ma S. Shindyapina A. V. Tikhonov S. Lee S. G. Bozaykut P. (2023). Distinct longevity mechanisms across and within species and their association with aging. Cell 186, 2929–2949.e20. 10.1016/j.cell.2023.05.002 37269831 PMC11192172

[B87] Watanabe K. Wilmanski T. Baloni P. Robinson M. Garcia G. G. Hoopmann M. R. (2023). Lifespan-extending interventions induce consistent patterns of fatty acid oxidation in mouse livers. Commun. Biol. 6, 768. 10.1038/s42003-023-05128-y 37481675 PMC10363145

[B88] Wilkinson J. E. Burmeister L. Brooks S. V. Chan C. C. Friedline S. Harrison D. E. (2012). Rapamycin slows aging in mice. Aging Cell 11, 675–682. 10.1111/j.1474-9726.2012.00832.x 22587563 PMC3434687

[B89] Williams E. G. Pfister N. Roy S. Statzer C. Haverty J. Ingels J. (2022). Multiomic profiling of the liver across diets and age in a diverse mouse population. Cell Syst. 13, 43–57.e6. 10.1016/j.cels.2021.09.005 34666007 PMC8776606

[B90] Wink L. Miller R. A. Garcia G. G. R. (2022). Rapamycin, Acarbose and 17α-estradiol share common mechanisms regulating the MAPK pathways involved in intracellular signaling and inflammation. Immun. Ageing 19 (8), 8. 10.1186/s12979-022-00264-1 35105357 PMC8805398

[B91] Xu S. Cai Y. Wei Y. (2014). mTOR signaling from cellular senescence to organismal aging. Aging Dis. 5, 263–273. 10.14336/AD.2014.0500263 25110610 PMC4113516

[B92] Yang S. Li M. Lian G. Wu Y. Cui J. Wang L. (2025). ABHD8 antagonizes inflammation by facilitating chaperone-mediated autophagy-mediated degradation of NLRP3. Autophagy 21, 338–351. 10.1080/15548627.2024.2395158 39225180 PMC11759624

[B93] Yang X. Zhong W. Cao R. (2020). Phosphorylation of the mRNA cap-binding protein eIF4E and cancer. Cell Signal 73, 109689. 10.1016/j.cellsig.2020.109689 32535199 PMC8049097

[B94] Yeh C. Y. Chini L. C. S. Davidson J. W. Garcia G. G. Gallagher M. S. Freichels I. T. (2024). Late-life protein or isoleucine restriction impacts physiological and molecular signatures of aging. Nat. Aging 4, 1760–1771. 10.1038/s43587-024-00744-7 39604703 PMC11672203

[B95] Zhang K. K. Burns C. M. Skinner M. E. Lombard D. B. Miller R. A. Endicott S. J. (2023). PTEN is both an activator and a substrate of chaperone-mediated autophagy. J. Cell Biol. 222, e202208150. 10.1083/jcb.202208150 37418003 PMC10327811

[B96] Zhao B. S. Roundtree I. A. He C. (2017). Post-transcriptional gene regulation by mRNA modifications. Nat. Rev. Mol. Cell Biol. 18, 31–42. 10.1038/nrm.2016.132 27808276 PMC5167638

