# Supplementary material for: The lifespan-extending MEK1 inhibitor trametinib promotes regulation of de novo lipogenesis enzymes by chaperone-mediated autophagy
Source: Front Aging. 2025 Jun 25;6:1621808. doi: 10.3389/fragi.2025.1621808 (PMC12237883; doi:10.3389/fragi.2025.1621808)
Supplement: Supplementary file 1 [file DataSheet1.pdf]

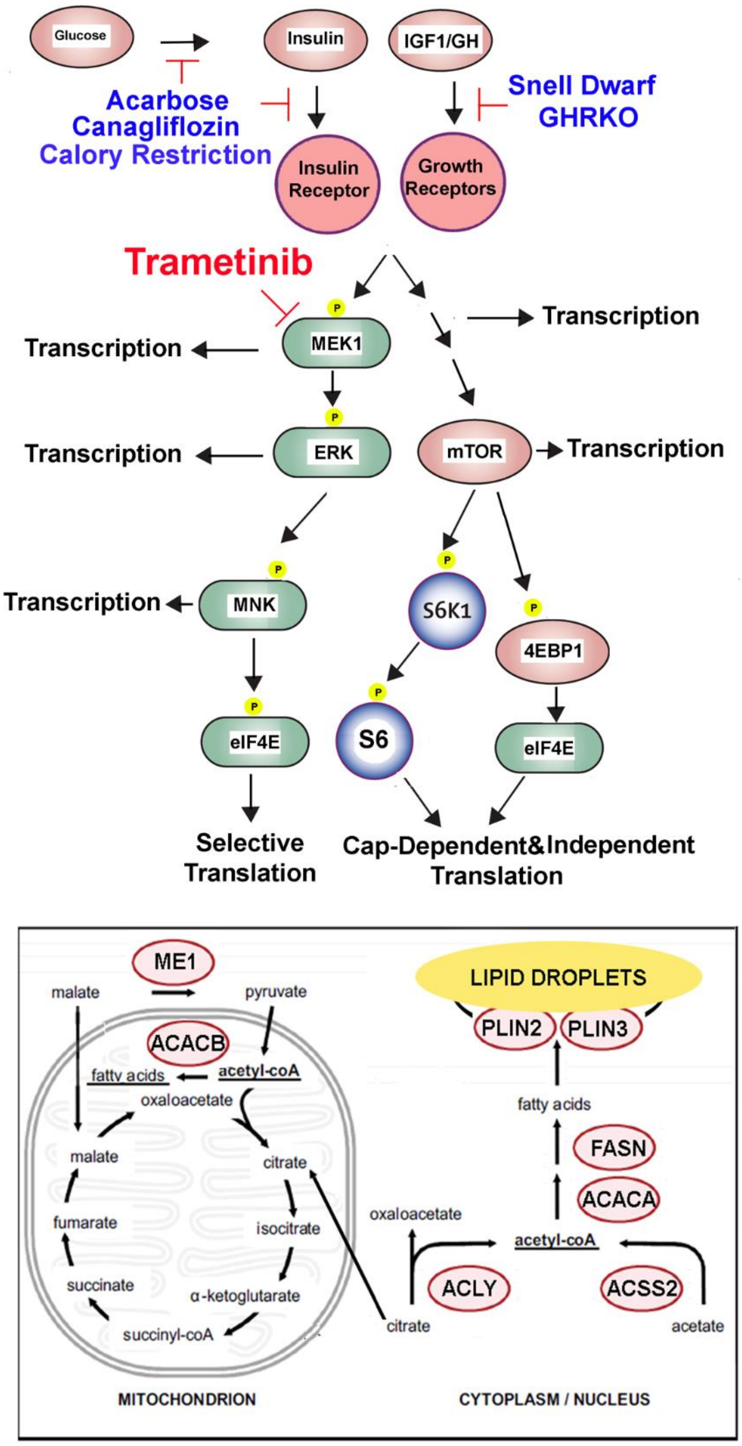

Figure S1. Insulin, IGF1, GH share common downstream signaling pathways that are downregulated by the effects of Aca, Cana, CR, and Snell or GHRKO mouse models. Both the mTORC1 and MEK1/ERK kinase pathways can affect transcription patterns, and the mTORC1 pathway is known to alter overall translation rates for protein synthesis. Our results show that the MEK1/ERK pathway, in addition to its documented effects on transcription, can modulate abundance of specific proteins by activation of CMA. Panel below show the location and function of the DNL enzymes.

F

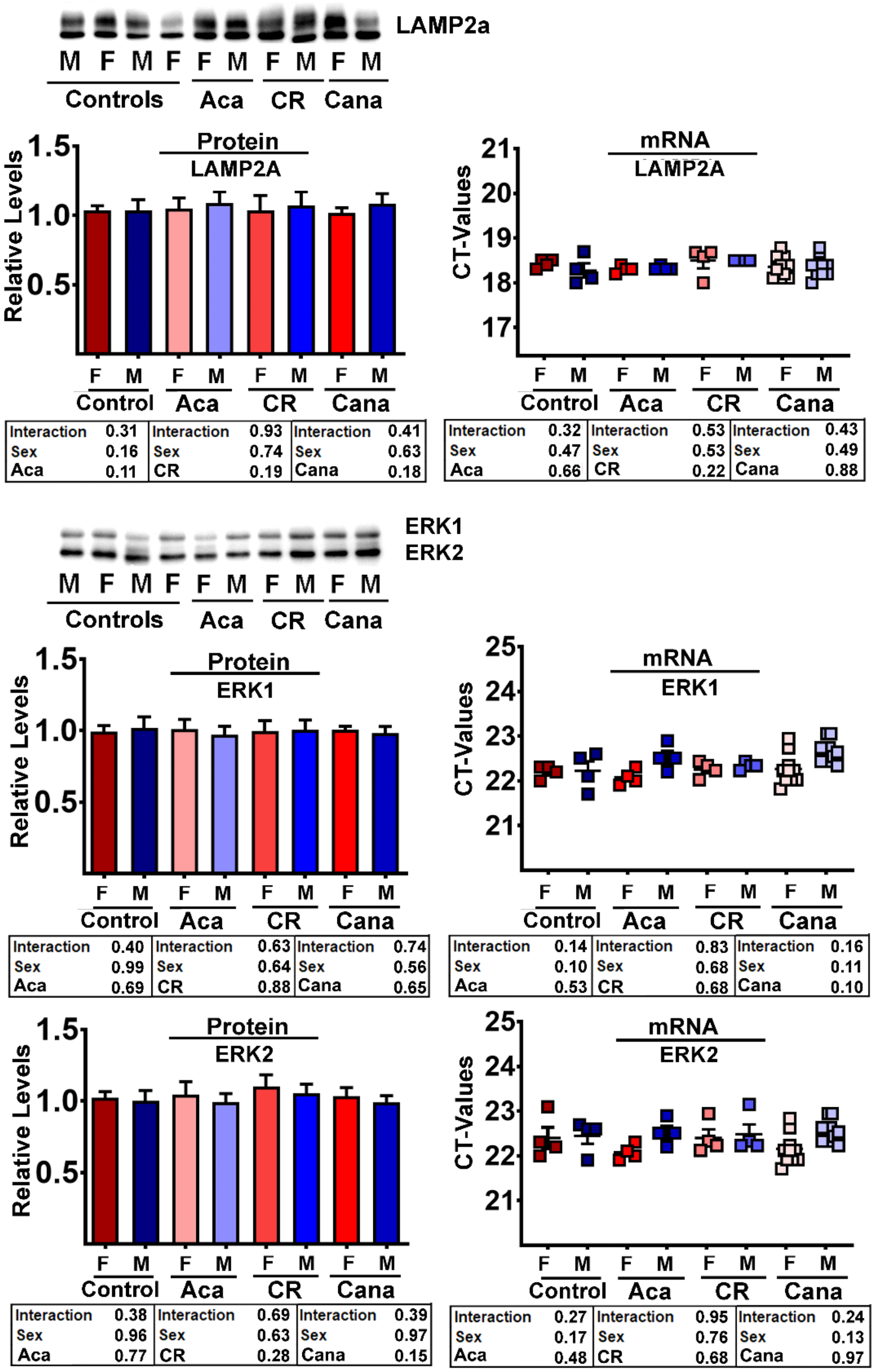

Figure S2A. Lamp2a, ERK1 and ERK1 were also used as internal controls for the normalization of western blots for Figure 2. There were no significant changes in the protein or mRNA levels.

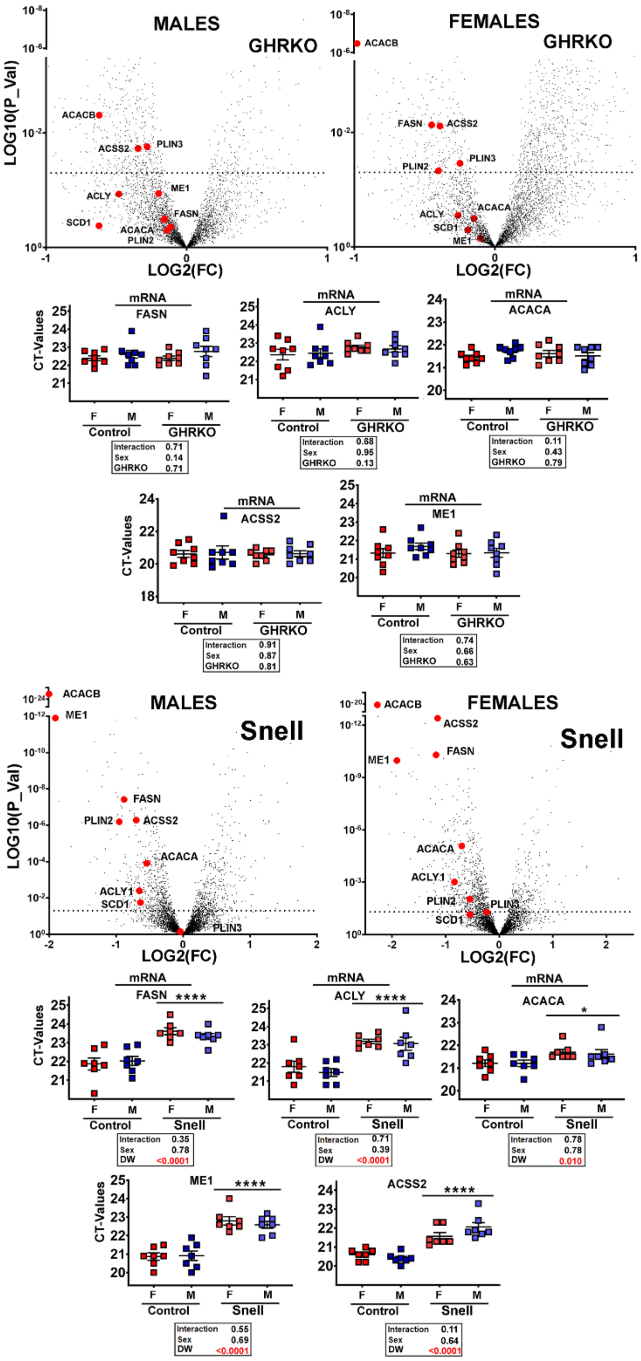

Figure S2B. Volcano plots represent the induced change in proteins (Log2 scale, horizontal axis) vs. corresponding p-value (log10 scale, vertical axis) for each of 4293 proteins measured in livers of 6-month-old GHRKO and Snell Dwarf mice, compared to littermate control samples. The nine red symbols indicate a set of DNL enzymes which were also shown in Figure 1. The scatterplots below show their mRNA levels with statistical results. There were no significant changes in the mRNAs of GHRKO, suggesting regulation in protein levels by a posttranscriptional mechanism. However, contrary to the GHRKO mice, mRNA in Snell Dwarf mice does show mRNA changes parallel to the protein declines.

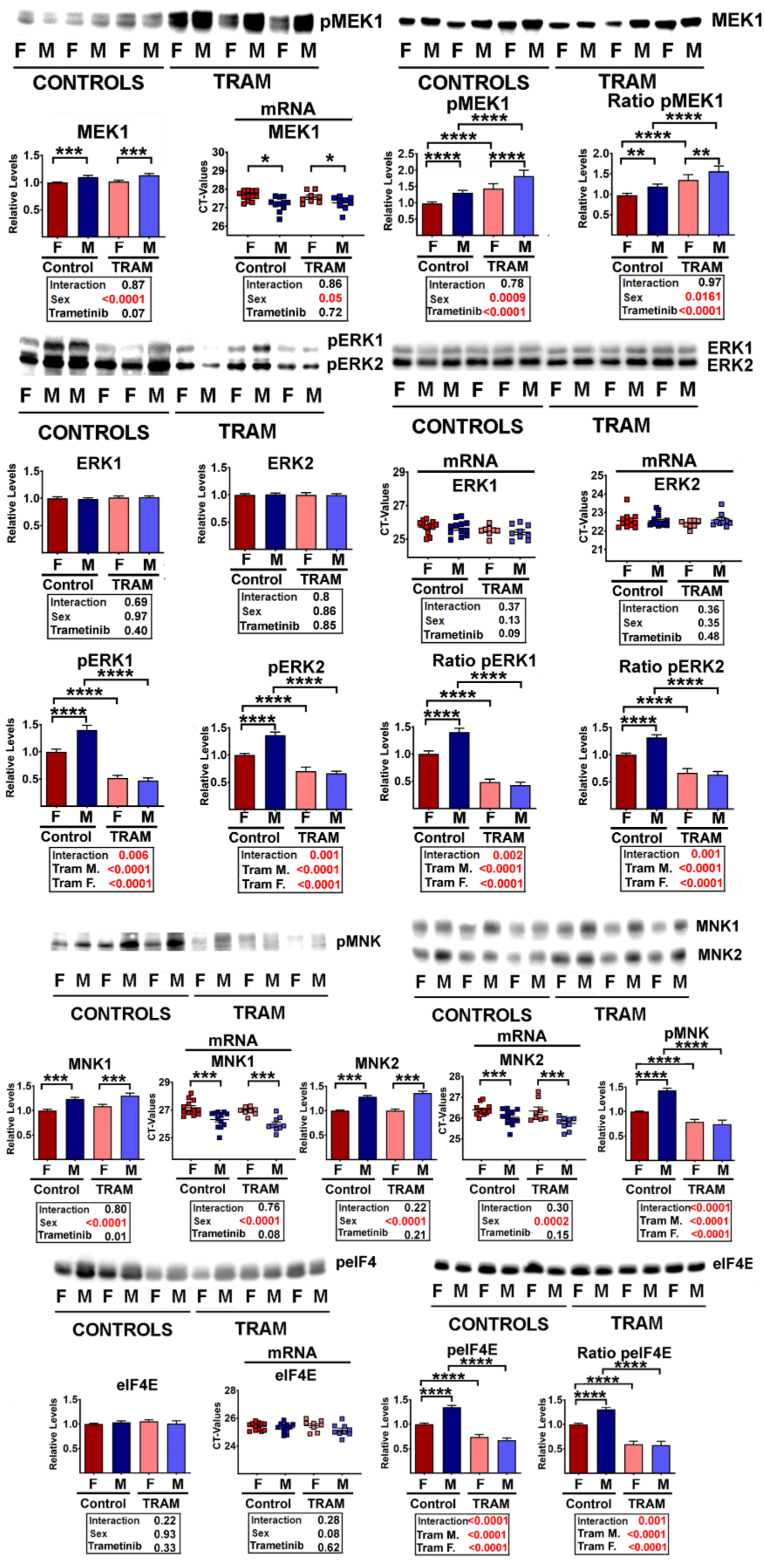

Figure S3A. In vivo, trametinib treatment as described in Figure 3 reduce MEK1-ERK-MNK-eIF4E signaling pathway without altering levels of the corresponding mRNA.

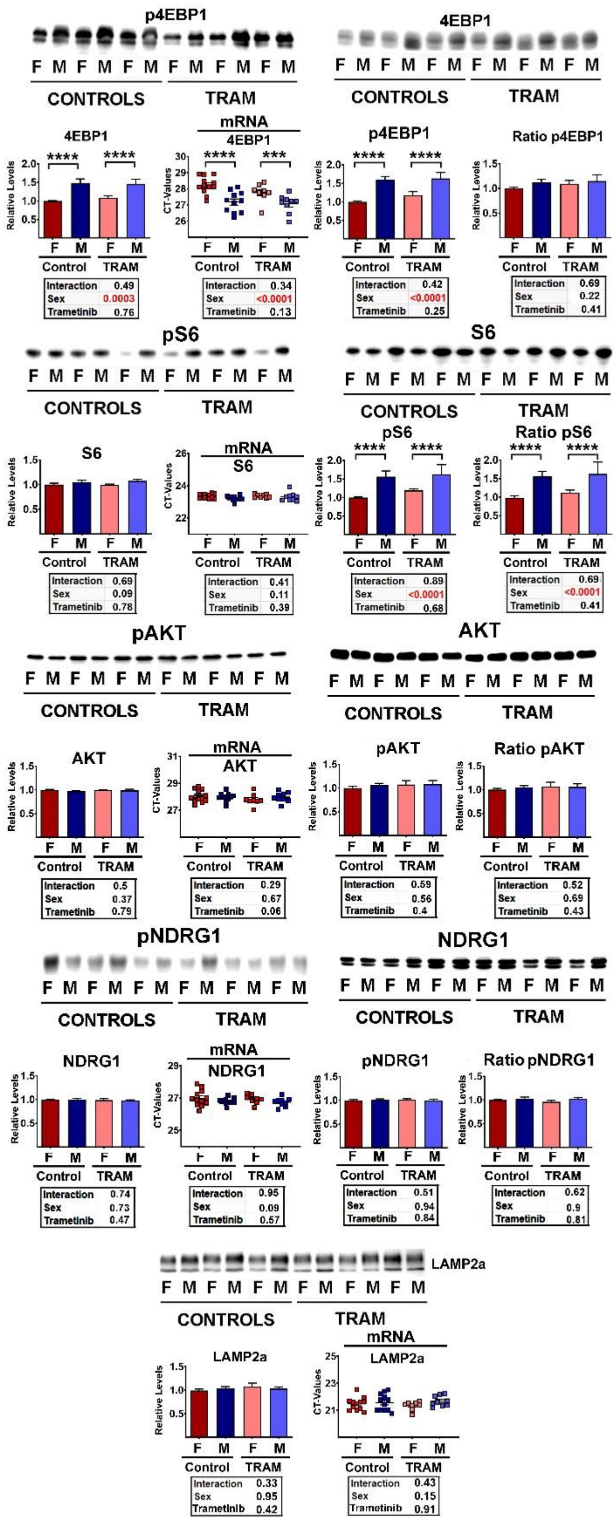

Figure S3B. In vivo trametinib treatments as described in Figure 3 do not alter mTORC1 (pS6 and p4EBP1) or mTORC2 (pNDRG and pAKT) signaling as well as LAMP2a levels or mRNA expression.

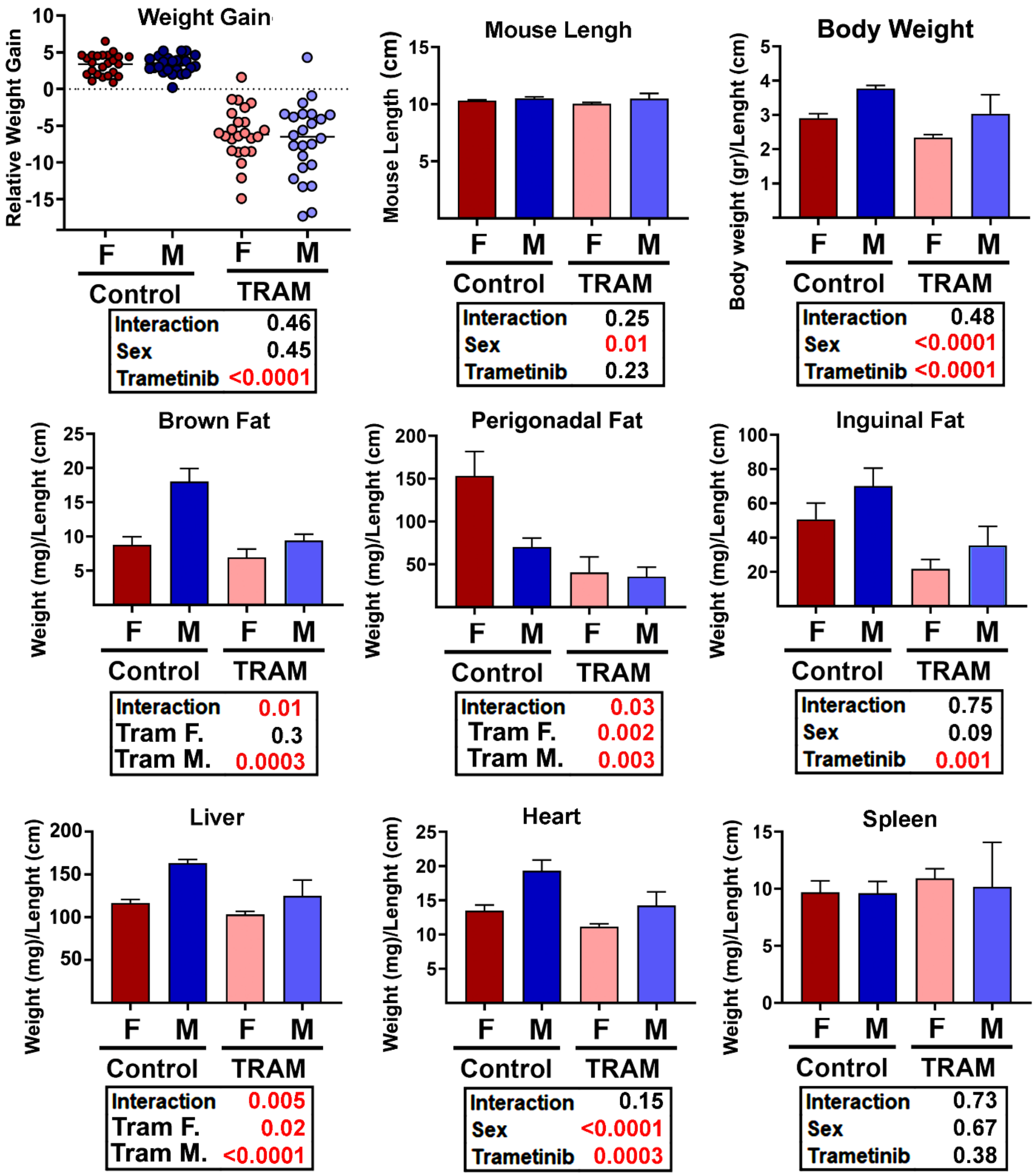

Figure S3C. Trametinib induces weight loss of fat and lean tissues in mice. (A) Weight change of each animal, over the course of the trametinib treatment, which indicates that trametinib induced weight loss. (B) Body length of the animals from the tip of the nose to the base of the tail, which did not differ based on trametinib treatment, but did differ based on sex. (C) Body weight of the animals at the time of dissection. (D) The weights of fat pads and organs at the time of dissection, presented both as absolute weight and as a percentage of total body weight. n = 10 animals for each of the four conditions. The same 40 animals were used for all displayed values. All data were analyzed by 2-factor ANOVA, and the results are shown below each plot.

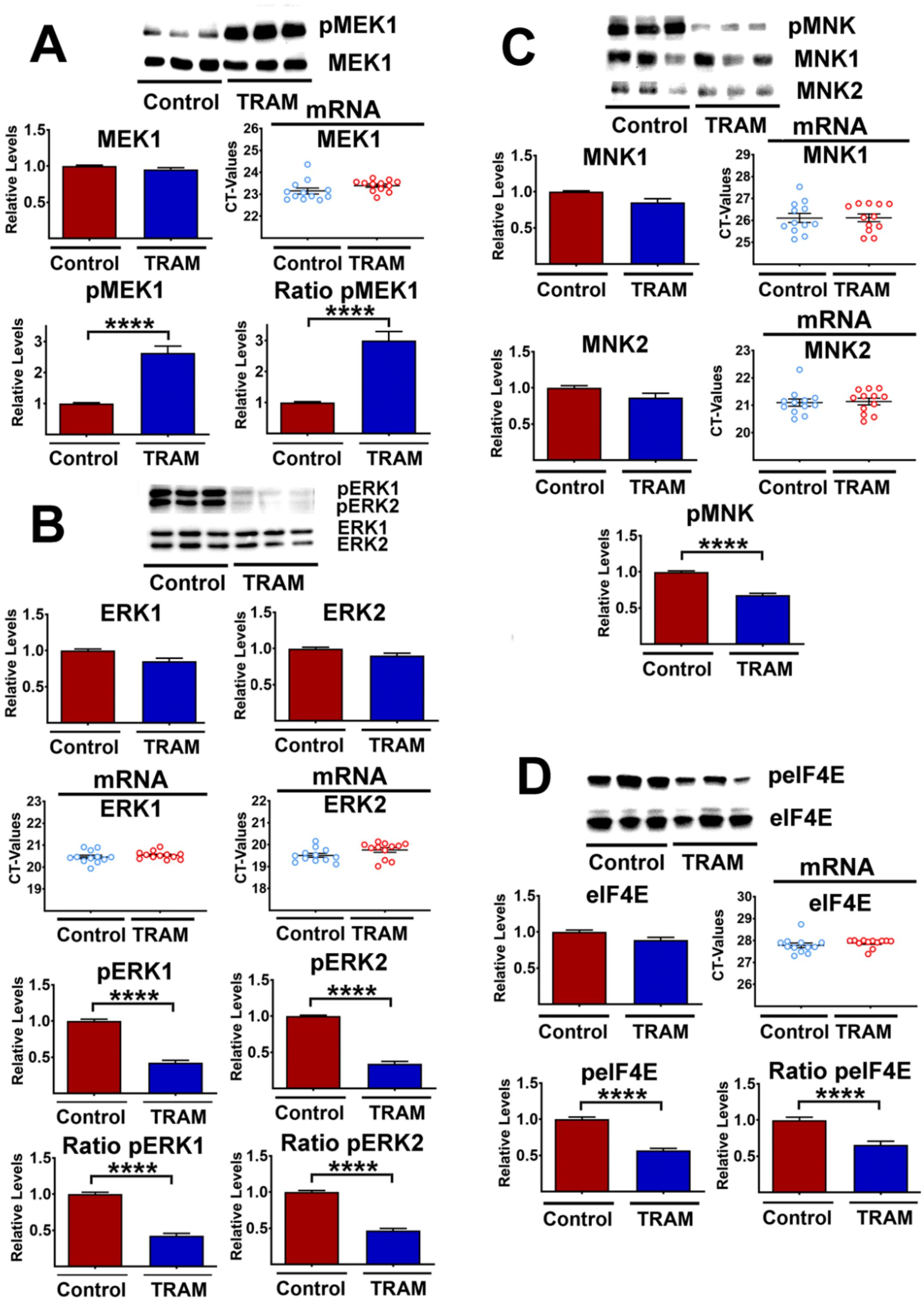

Figure S4A. AML cells treated with trametinib as described in Figure 4 reduces activity of enzymes in the MEK1-ERK-MNK-eIF4E signaling pathway without altering their mRNA levels.

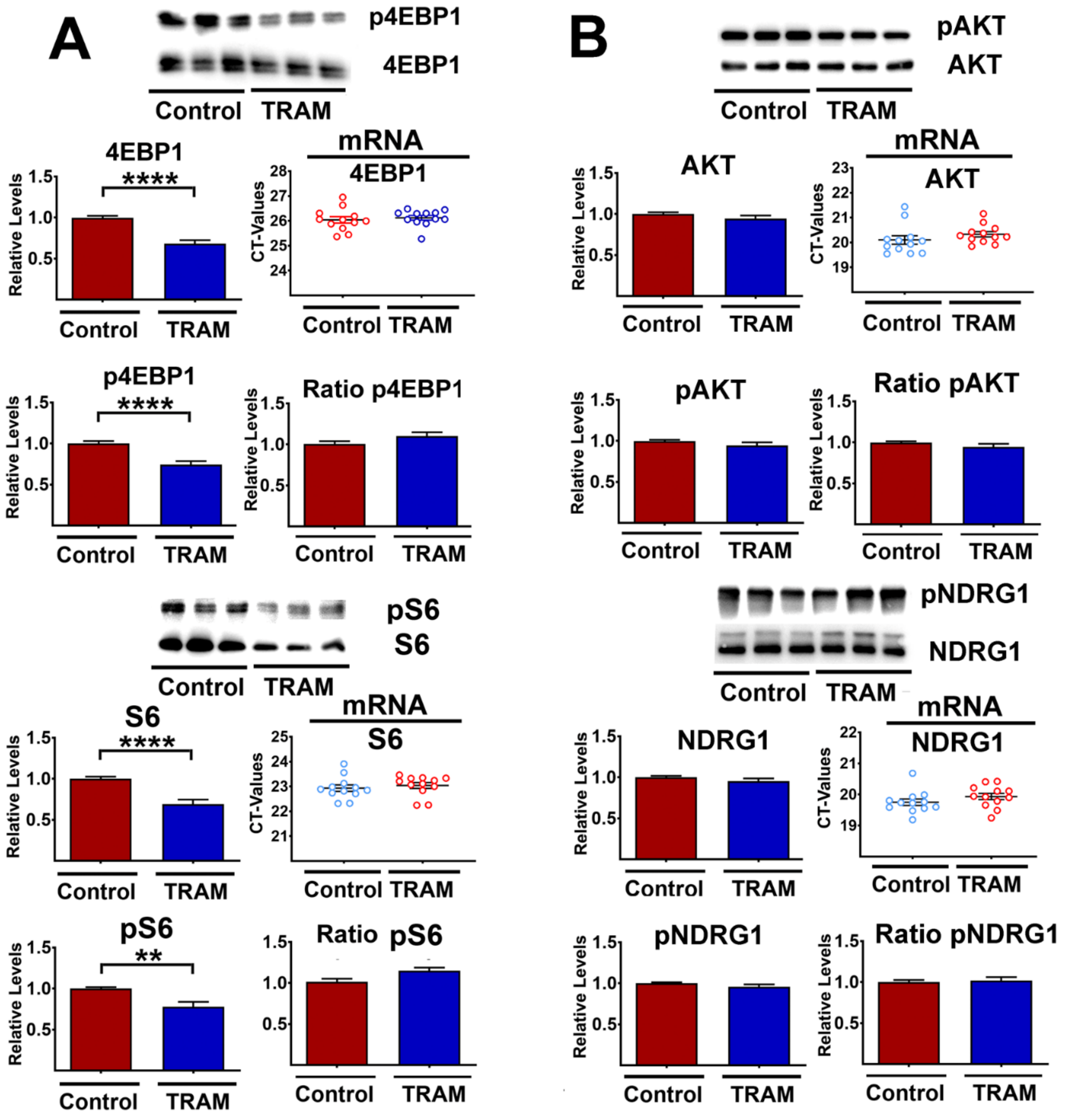

Figure S4B. Tram treatment of AML cells, as in Figure 4, leads to small but significant declines in the level of phosphoproteins pS6 and p4EBP1. The level of these two proteins also declines in Tram-treated cells, and the ratio of phosphoprotein to total protein is therefore unchanged. The change in total S6 and 4EBP1 protein is not accompanied by parallel changes in their mRNA. Tram has no effect on total amount of phosphoprotein level for NRDG1 and AKT (mTORC2).

Table S1

Supplementary Table 1

qRT-PCR probes

Gene Name	Name	Reference Sque	Forward Probe	Reverse Probe
Fatty acid synthase (Fasn)	FASN	NM_007988.3	CCCCTCTGTTAATTGGCTCC	TTGTGGAAGTGCAGGTTAGG
Acetyl-Coenzyme A carboxylase alpha (Acaca)	ACACA	NM_133360.3	AAGGCTATGTGAAGGATGTGG	CTGTCTGAAGAGGTTAGGGAAG
Acetyl-Coenzyme A carboxylase beta (Acacb), transcript variant 2	ACACB	NM_133904.3	AGTCTTCCGTGCCTTTGTAC	TTCTGCAAACTCATCCCTCG
Perilipin 2 (Plin2), transcript variant 1	PLIN2	NM_007408.4	GGATAAGCTCTATGTCTCGTGG	GTCTGGCATGTAGTCTGGAG
Perilipin 1 (Plin1), transcript variant 1	PLIN1	NM_175640.2	ACAGACACAGAGGGAGAGG	AGTGTTCTGCACGGTGTG
Acyl-CoA synthetase short-chain family member 2 (Acss2)	ACSS2	NM_019811.3	ACCAGTTAAGAGGCCATGTC	GTACAAGATGAAGAGTGGGTCC
ATP citrate lyase (Acly), transcript variant 1	ACLY	NM_001199296.1	ACCAGTTAAGAGGCCATGTC	GTACAAGATGAAGAGTGGGTCC
Malic enzyme 1 (Me1)	ME1	NM_008615.2	ATCCATGACAAAGGGCAC	ATCCCAATTACAGCCAAGGTC
AKT1 (RACALPHA) AKT serine/threonine kinase 1 (Akt1)	AKT	NM_009652.4	GCCCTCAAGTACTCATTCCAG	ACACAATCTCCGCACCATAG
Mitogen-activated protein kinase kinase 1 (Map2k1)	MEK1	NM_008927.5	TTCCCGGCTGCAAGATG	CTTCTGCAAGGCCTCCAG
Mitogen-activated protein kinase 3 (Mapk3)	ERK1	NM_011952.2	GTTATAGGCATCCGAGACATCC	GTAGAGGAAGTAGCAGATGTGG
Mitogen-activated protein kinase 1 (Mapk1)	ERK2	NM_011949.3	GGCAGGTGTTCGACGTAG	AGTAGGTCTGGTGCTCAAAAG
MAP kinase-interacting serine/threonine kinase 1 (Mknk1)	MNK1	NM_001355174.1	TGTGACTTTGACTTGGGCAG	GTCATAGAAAGTAGCCTCGTCC
MAP kinase-interacting serine/threonine kinase 2 (Mknk2)	MNK2	NM_021462.5	GGGAGGTGGAGATGCTGTA	CTATGGATGTGGCTTAGGATGG
Eukaryotic translation initiation factor 4E (Eif4e), variant 1	EEF4E	NM_007917.4	TTACAGTCCTTACCACAGCAC	GTTCCACAGTCGCCATCTTAG
Ribosomal protein S6 (Rps6)	S6	NM_009096.3	GCAAACTCTACCTCATCCTGG	GCTAGTGTGATCTCTGCCAG
Eukaryotic translation initiation factor 4E binding protein 1 (Eif4ebp1)	4EBP1	NM_007918.3	CGGAAGATAAGCGGGCAG	CAGTGTCTGCCTGGTATGAG
Eukaryotic translation initiation factor 4E (Eif4e), variant 1	NDRG1	NM_008681.2	CGAGAGCTACATGACGTGGA	AAGAGGGGGTTGTAGCAGGT
Beta-Actin	B-Actin	NM_007393	CTAAGGCCAACCGTGAAAAG	ACCAGAGGCATACAGGGACA

Sources of antibodies

Gene Name	Name	Cat#	Source
Acetyl-Coenzyme A carboxylase alpha	Acaca	4190	Cell Signaling Technology
ATP citrate lyase	Acly	40793	AbCam
Fatty acid synthase	Fasn	22759	AbCam
Perilipin 2	Plin2	A20843	AbClonal
Perilipin 1	Plin1	A4758	AbClonal
Acyl-CoA synthetase short-chain family member 2	Acss2	3658	Cell Signaling Technology
Malic enzyme 1	Me1	ab97445	AbCam
AKT (Pan) serine/threonine kinase	Akt	4691	Cell Signaling Technology
Phospho-AKT at S473	pAkt	4060	Cell Signaling Technology
Erk kinase 1 or MEK1	Mek1	12671	Cell Signaling Technology
Phospho-MEK1 at S217/221	pMek1	9154	Cell Signaling Technology
p44/42 MAPK (Erk1/2)	Erk1/2	4695	Cell Signaling Technology
Phospho-p44/42 MAPK at Thr202/Tyr204	pErk	4376	Cell Signaling Technology
Mitogen-activated protein kinases 1	Mnk1	2195	Cell Signaling Technology
Mitogen-activated protein kinases 2	Mnk2	17354	ProteinTech
Phospho-Mitogen-activated protein kinases at T197/202	pMnk	700242	ThermoScientific
Eukaryotic translation initiation factor 4E	4Ebp1	9644	Cell Signaling Technology
Phospho-eukaryotic translation initiation factor 4E atT37/46	p4Ebp1		Cell Signaling Technology
Ribosomal protein S6 (Rps6)	S6	2217	Cell Signaling Technology
Phospho-ribosomal protein S6 (Rps6) at S235/236	pS6	4857	Cell Signaling Technology
N-myc downstream regulated gene 1	Ndrg1	9408	Cell Signaling Technology
Phospho N-myc downstream regulated gene 1 at T346	pNdrg1	5482	Cell Signaling Technology
b-Actin	B-Actin	8457	Cell Signaling Technology
Lysosome-associated membrane protein 2 A	LAMP2A	125068	AbCam
Eukaryotic translation initiation factor 4E	Eif4e	2067	Cell Signaling Technology
Phospho-eukaryotic translation initiation factor 4E atS209	pEif4e	p00135	Boster
